# Living archival practice and the choreographical navigations: Encounters and approaches with other-than-human persons

**DOI:** 10.12688/openreseurope.17447.1

**Published:** 2024-08-07

**Authors:** Shuntaro Yoshida, Alex Viteri Arturo, Catalina Fernandez, Maharu Maeno, Mapped to the Closest Address, Jun Yamaguchi

**Affiliations:** 1 Waseda University, Shinjuku, Tokyo, Japan; 2 Berlin University of the Arts, Berlin, Berlin, Germany; 3 CUNY The Graduate Center, New York, New York, USA; 4 Mapped to the Closest Address, Berlin, Berlin, Germany; 5 Weißensee Kunsthochschule Berlin, Berlin, Berlin, Germany; 6 Tokyo City University, Setagaya, Tokyo, Japan; 7 Musashino Art University, Kodaira-shi, 1588557, Japan

**Keywords:** Other-than-human persons, malfunction, disobedient movement, choreography

## Abstract

This article delves into the collaborative work of the interspecies dance collective, Mapped to the Closest Address (MaCA), focusing on our living archival practice and exploration of choreography with other-than-human persons. Through encounters with various species and environments, MaCA seeks to shift anthropocentric perspectives, interrogate their orientation towards modernity and coloniality, and question their understanding/administration/entanglement/devotion of, with, and to nature. The collective’s journey, from a digital residency during the COVID-19 pandemic to site research, installations, and performance at the Echigo-Tsumari Art Triennale 2022, is documented and analyzed.

The collective’s collaborative process involves relinquishing control to allow for the emergence of disobedient movements and the exploration of choreography from the perspective of other-than-human persons. This includes encounters with kudzu vines and mountains, weaving their movements and patterns into performances and installations. The article discusses the immersive performance “Turn Off the House Lights,” in which MaCA integrates stories from local communities with gestures inspired by the landscape.

Through our living archival practice, MaCA aims to transmit a collective memory of interactions among organisms and environments and highlight the interconnectedness of humans and the other creatures of the Earth. The article reflects on the significance of choreography beyond human-centric notions, emphasizing the emergent forms of ecological performance and the dissolution of boundaries between human and non-human realms.

Drawing on interdisciplinary perspectives including dance, visual art, and theatre, MaCA’s work exemplifies a cross-disciplinary approach to expressing the choreography of other-than-human persons. This approach not only presents audiences with immersive experiences but also responds to the future ecosystem through artistic exploration. Ultimately, MaCA’s living archival practices contribute to awareness of the collective lives of other-than-human persons and offer insights into navigating our enmeshment with the natural world.

Human beings are part of nature.

( Echigo-Tsumari Art Field, n.d.)

## Introduction

**Figure 1.**
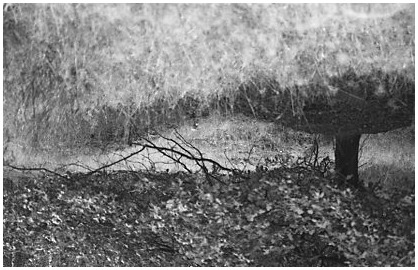
Mushroom field surrounded by forest at El Arenero Yumita. 35mm black-and-white film. Photo by Alex Viteri Arturo.

From 2019 to 2022, Mapped to the Closest Address (MaCA) was an interspecies dance collective composed of four human animals (Colombian performance maker Alex Viteri Arturo, Colombian sound/light designer Catalina Fernandez, Japanese visual and performance artist Maharu Maeno, and Japanese dancer/choreographer Shuntaro Yoshida) and a cat, Violeta. Through choreographic practices, the humans in the group sought to interrogate their orientation towards modernity and coloniality, question their understanding/administration/entanglement/devotion of, with, and to nature, and shift their anthropocentric perspectives. ^1^ Collectively, we sowed practices to enter into contact with other-than-human persons. We used various devices to record our encounters and crafted multimedia social spaces to share our archive ( Figure 1). The following discussions of our collaboration explore the history of our encounters.

This article considers our research-creation, from our recollections of a digital residency in 2020 during the COVID-19 pandemic to site research, installation, and performance at the Echigo-Tsumari Art Triennale 2022 in Niigata. We archived the movement of other species and collectively translated the awareness of local ecosystems within planetary and seasonal processes into our choreography. ^2^ In other words, through the accumulation of living archives, our collective memory of interactions among organisms and environment navigating the other-than-human persons' choreography ^3^ in the context of multispecies ethnography and animistic approaches in art creations. The detailed examination of these perspectives in “Turn Off the House Lights” aligned with emergent ecological performances. This sound-dance performance was held at the Echigo-Tsumari Art Triennale in 2022. Our creation process, involving other species, has focused on the interconnectedness of humans and non-humans. However, in this article, we turn our attention to the approaches to and significance of the choreography of other-than-human persons.

To explore our living archival practice, this article draws on dance and theatre writer Maša Radi Buh’s notion of ecological choreography, foregrounding not only the ecological experience of awareness but also the future ecosystem, imagining a post-apocalyptic time without humans through a geolocational sound walk that she mentioned. Furthermore, the choreography values “a (utopian) idea of a blurring of the distinction between (the value of) human and non-human creatures appears.”( Buh, 2022, 48) This article examines our ecological choreography strategies via malfunction and disobedient movement, revealing how such movements expose the dissolution of the border between human and non-human. Specifically, we aim to act out the transmission of our collective memory of landscape by encountering other-than-human persons. Our creation then shifts to tuning to local ecologies, and we encounter the notion of “disobedience” through other-than-human persons’ choreography. To illuminate the experience of heightened ecological awareness generated by disobedient movement, we speculate on the choreographic navigations.

## Malfunctioning video and non-human choreography: Open Forest Launch, a digital residency (Frankfurt an der Oder/Kōto-ku, Tokyo)

MaCA tended two vegetable gardens: a family’s backyard garden in Hyogo (Maharu and Shuntaro) and a *Schrebergarten* in Frankfurt an der Oder (Catalina, Alex, and Violeta). We named the latter “Arenero Yumita” as a tribute to American scholar of Chicana feminism Gloria Anzaldúa and Catalina’s mother Luz Marina Giraldo “Yumita.” This represented a wishful naming for our queer family sanctuary and place to encounter and learn from other-than-human beings. ^4^ Through MaCA’s horticultural practice and research, we strived to cultivate a heightened awareness of planetary and seasonal processes and translated them into our notion of choreography. ^5^

**Figure 2.**
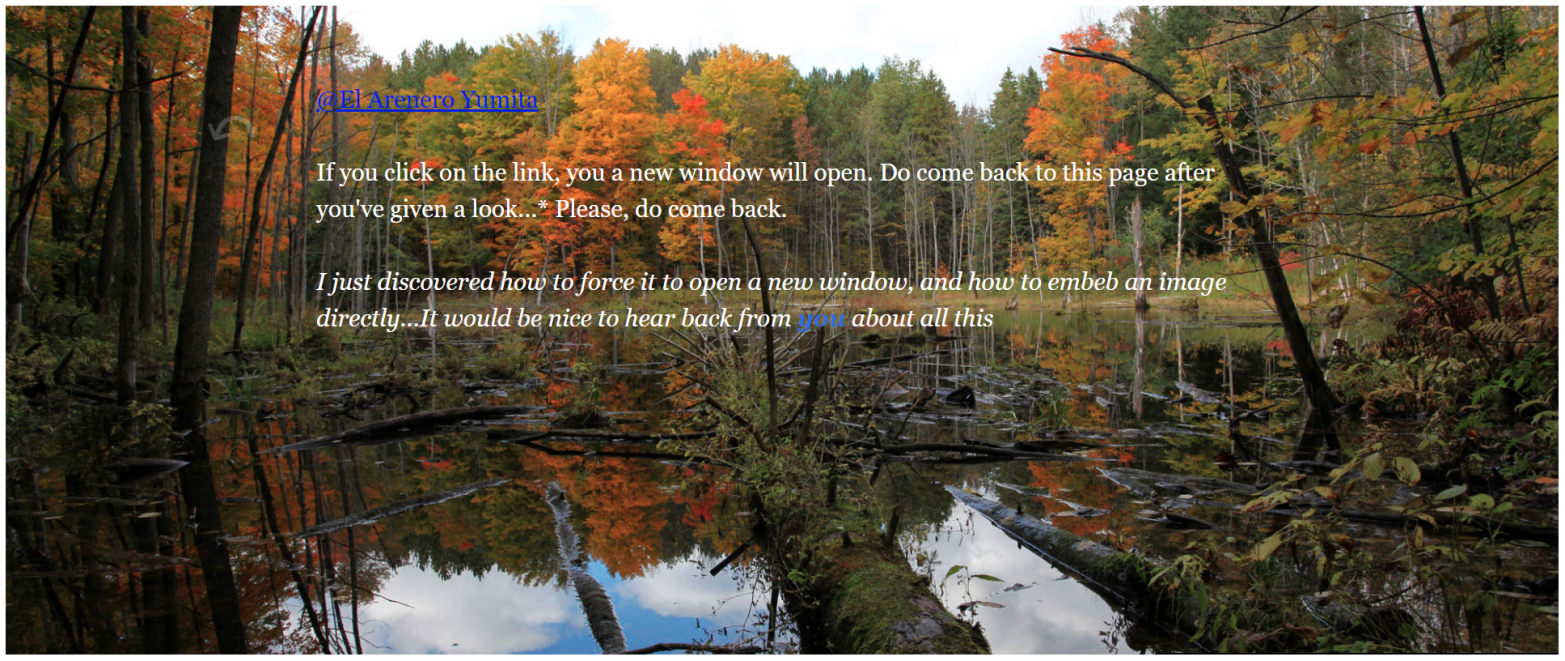
Screenshot of “Open Forest Launch” in progress. Photo by Catalina Fernandez.

Unable to travel during the COVID-19 pandemic, we worked remotely, devising a digital residency to exchange the sensory experiences of our respective locations. Hosted by the Saison Foundation, we investigated various ways to relate with non-human beings. ^6^ Our practices combined readings on non-human personhood with gardening. This led to “Open Forest Launch,” a collection of videos documenting our exchanges uploaded to a digital world still in progress ( Figure 2). ( Mapped to the Closest Address, 2020a)

**Figure 3.**
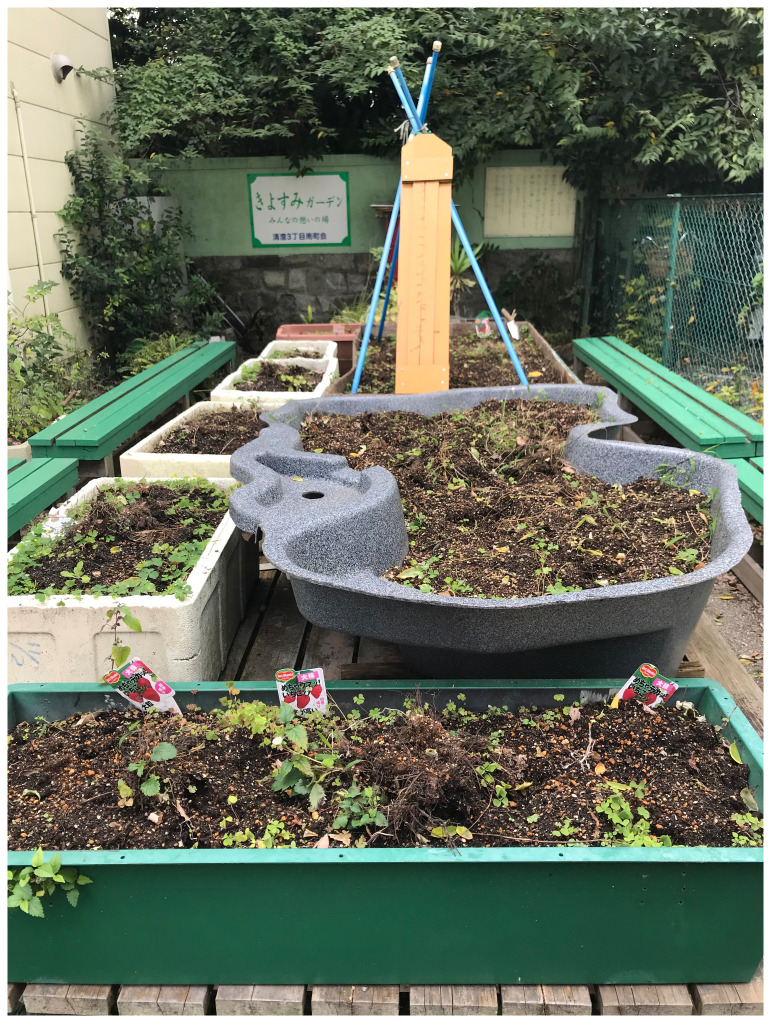
Kiyosumi community garden in Kōtō-ku. Photo by Shuntaro Yoshida.

**Figure 4.**
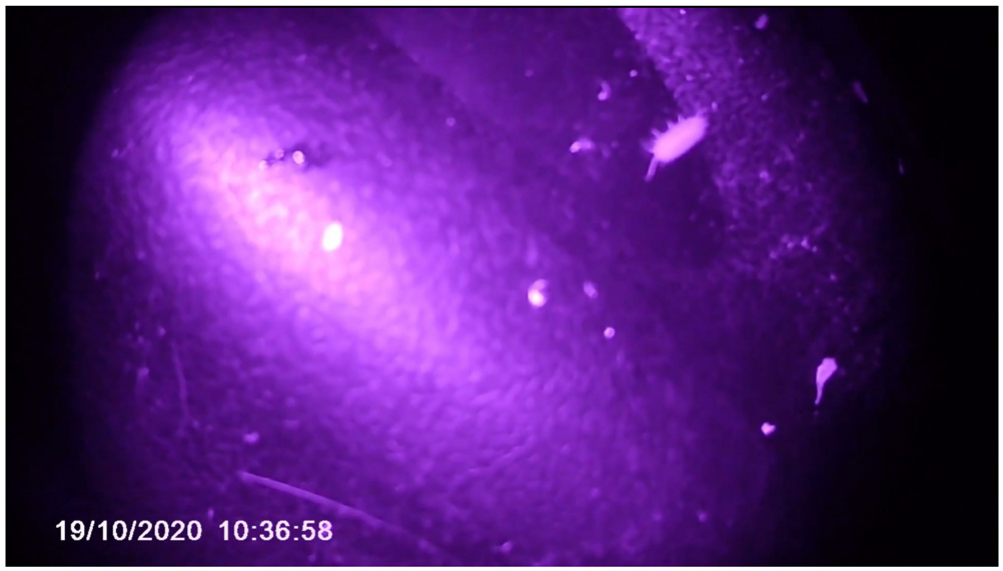
Scene from “Open Forest Launch_Video Diaries.” Captured by Violeta and edited by Catalina Fernandez.

**Figure 5.**
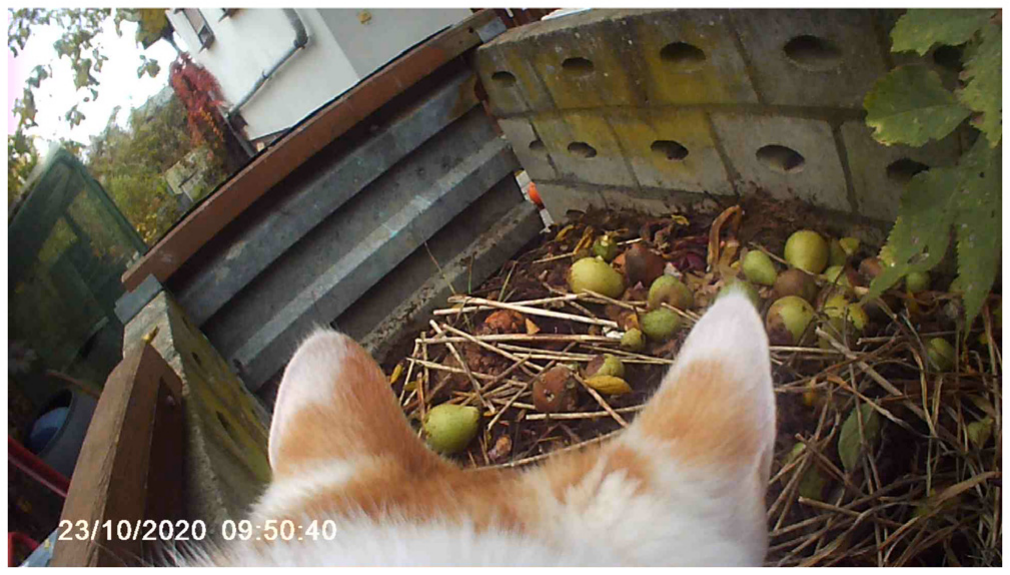
Scene from “Open Forest Launch_Video Diaries.” Captured by Violeta and edited by Catalina Fernandez.

In the video diaries, we witness the slow growth of vegetables, join the Kiyosumi community garden in Tokyo ( Figure 3 and Figure 4), and glimpse the depths of the Hellenesee, a sinking lake on the Germany-Poland border. ( Mapped to the Closest Address, 2020b) We sought to draw attention to diverse human and non-human players to dislocate our anthropocentric standpoint and explore from other points of view the many worlds within gardens, forests, and lakes. A cat, Violeta joined the collective as a non-human videographer, and her material became a fundamental part of the work. She made several field trips with a miniature spy camera attached to her back. Violeta’s footage served as one of the living archives that revealed and documented how she moved. Similarly, we reimagined her life and world through her actions, such as following her gaze and climbing up trees, and communicating with crows along with Violeta ( Figure 5). In her video, we noticed the impossibility of translating concepts between the humans and the non-humans, so we decided to cohabit with non-human movement.

The camera captured a view from a much lower position than a human’s camera would be placed and produced seemingly chaotic images. We were exploring with the camera, which was bound by her pace and the fact that she was on four legs. Accepting the malfunction of the video was also a way of relinquishing mastery over the final art product. As the camera had been given to Violeta, human choreography committed to her and there was no way we could control her moves: where she went, how long she decided to stay in a spot, and how fast she was. We relinquished the mastery over the video capture to Violeta. The video diary, including her perspectives, was also an attempt to take our thinking past human phenomena to a place beyond the human. Thus, the footage served as a portal that allowed us to escape, although briefly, from our human perspective.

## Kudzu and mountains as other-than-human personhood: Clumsy-seeming mountains and site research (Tokamachi)

**Figure 6.**
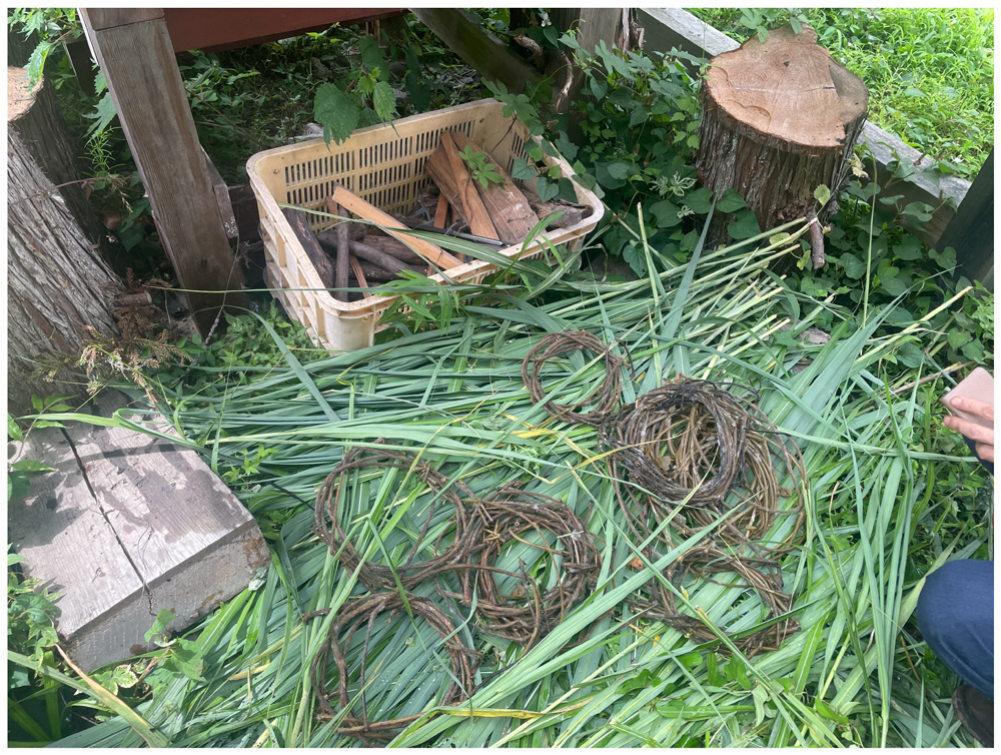
Maharu Maeno and Jun Yamaguchi cutting kudzu. Photo by Shuntaro Yoshida.

**Figure 7.**
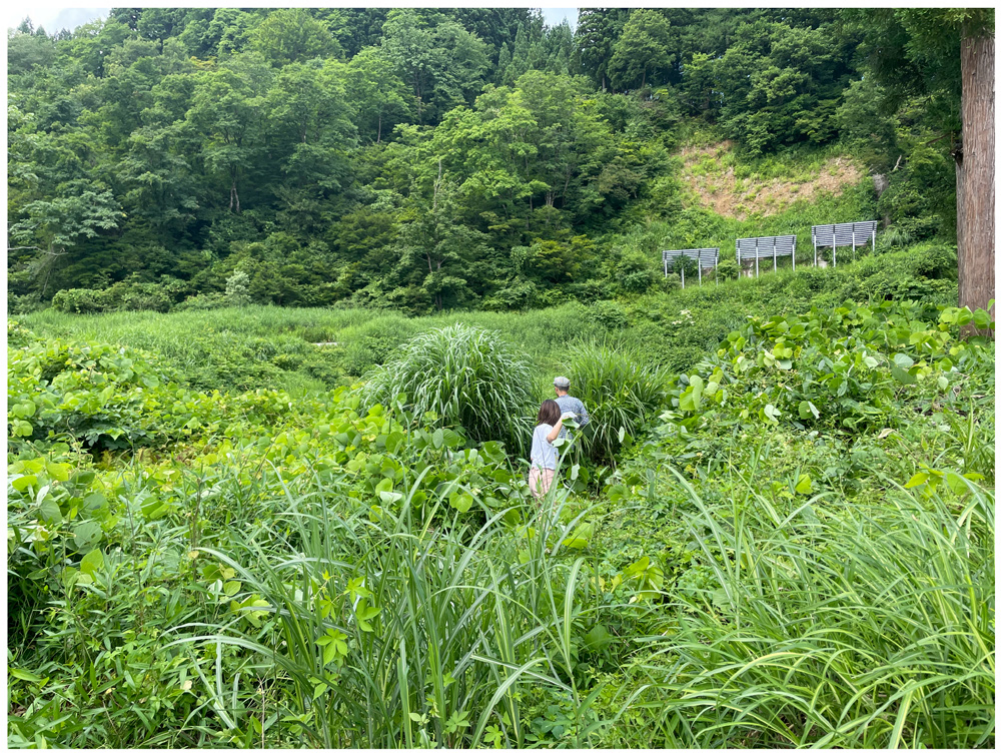
Kudzu fermentation (Pictured: J. Yamaguchi and M. Maeno). Photo by Shuntaro Yoshida.

MaCA received a second invitation, this time to participate in the Echigo-Tsumari Art Triennale (ETAT). ^7^ We traveled several times to the province, visited our assigned space, and planned for field research. The city of Tokamachi is surrounded by mountains, which are called *satoyama*. ^8^ Prior to the opening of the festival, Shuntaro, Maharu, and architect Jun Yamaguchi ^9^ stayed at Yamaneko Guest House to explore the three nearby mountains: Akiba, Gongen, and Takaba. They encountered and collected kudzu vines from up in the hills around the guest house ( Figure 6). While craftsmen of the ancient Jōmon periods (c. 14000 to 300 BCE) utilized kudzu for fibers and cloth, few such techniques remain. Today, the vine is deemed invasive and categorized as a weed. However, after engaging in processes like boiling, fermenting ( Figure 7), and river-washing to gauge its durability, we shifted our perspective on kudzu. We recognized our intricate connection with kudzu and choreographed movements reflecting this entanglement. We learned from kudzu’s weaving patterns, integrating them into our mountain portraits for the installation. We followed the patterns as if dancing, by handweaving the dried leaves. The choreographic score comprised, in Jun’s words, “covering,” “spreading in search of the sun,” and “intertwining.” These extended non-human choreographies were knitted again how plants live and move on the land, and we noticed their life compositions. ^10^ We stayed close to kudzu in terms of our embodied knowledge as inheritors of their movements.

While contemplating the mode of existence of kudzu and the functional workings of these life forms, we explored the choreography of the intelligence of plants through the practice of listening to their rhythms and movements. Their tips were growing very vigorously in the summer. Listening to and cohabiting with their movements, we unravel the composition of kudzu. Becoming attentive to kudzu also leads to an acceptance of the mountains, the “Earth beings” which allow us to engage in a sensory dialogue with organic matter. (Cadena, 2015, 105) ^11^

While researching the Akiba 秋葉, Gongen 権現, and Takaba 高場 mountains, which surround the exhibition site, we encountered multispecies stories. At the summit of Akiba mountain stands a shrine dedicated to Akiha Sanjakubo 秋葉三尺坊 or Akiha Gongen 秋葉権現, which is a manifestation of the Buddha as a Shinto god of fire protection appearing as a long-nosed Japanese goblin called a *tengu* 天狗 (a divine being in Japanese folklore). During the Edo period (1603–1867), Akiha Sanjakubo was widely revered as having control over fire. Akihabara 秋葉原, the neighborhood of Tokyo where we bought electric parts for our installation, is renowned globally for anime and subculture, and derives its name from the Akiha Gongen. Legend has it that the technique for extracting thread from kudzu was developed by a Shugendo practitioner in Kakegawa, Shizuoka prefecture, not far from another Akiha mountain. ( Fukatsu, 2008, 41) To this day, kudzu cloth continues to be crafted in Kakegawa. The mountains transmitted knowledge to us, and we were tuning in to their rhythms rather than the other way around.

**Figure 8.**
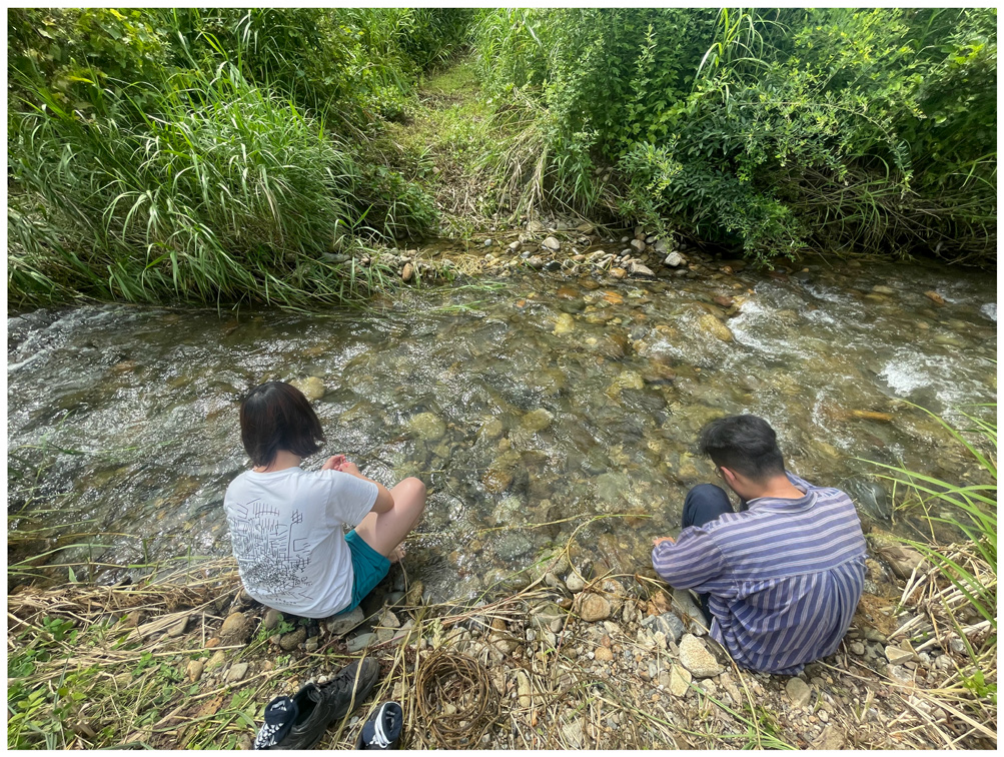
Washing kudzu (Pictured: J. Yamaguchi and M. Maeno). Photo by Shuntaro Yoshida.

**Figure 9.**
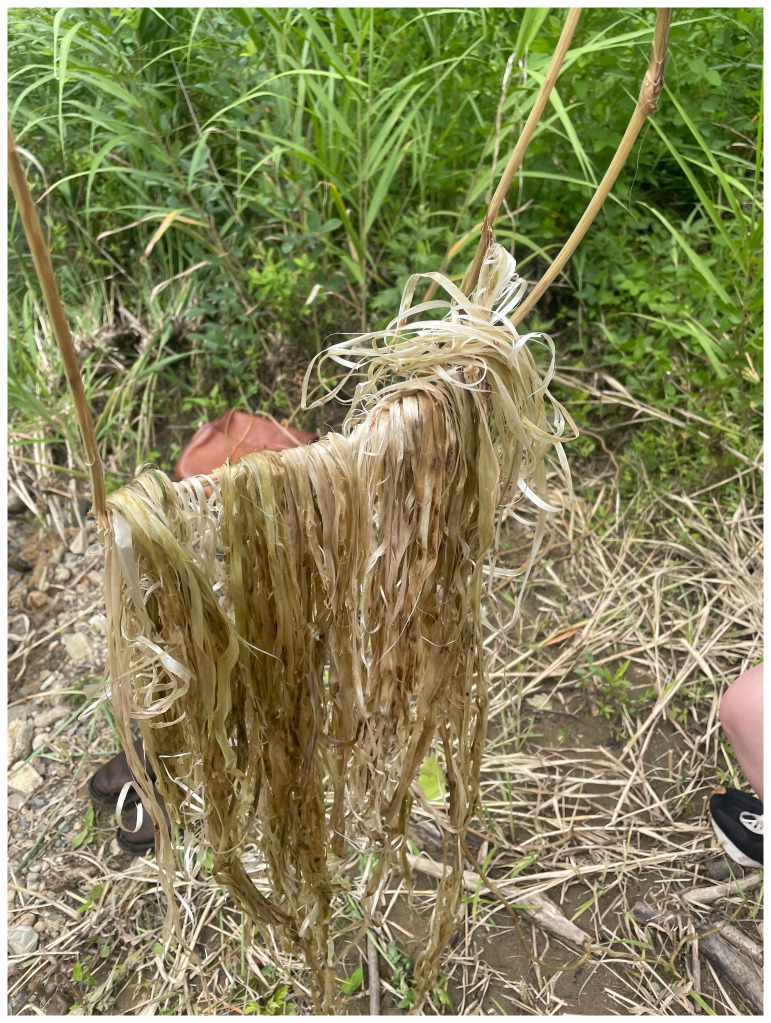
Kudzu fiber. Photo by Shuntaro Yoshida.

Through kudzu movements and our gestures (boiling, fermenting, and river-washing) which render kudzu into fiber, we encountered a composition of kudzu which tells of the loss of craft skills in an era of modernization ( Figure 8 and Figure 9). When we translate the kudzu movement into dance scores, the witness of kudzu links the memory of local territories and the presence of *satoyama*. *Satoyama* attunes us to the rhythm of kudzu in Echigo’s ecosystem and debates the symbiosis between humans and non-humans. However, we do not only demonstrate that “human beings are part of nature,” but also that kudzu and Akiba mountain in their other-than-human personhood echo with the past and future of the life cycle and resist human control – we watch the clumsy-seeming mountains.

## Navigation of the living archives and ecological considerations: *Clumsy-Seeming Mountains*, the installation (Tokamachi Snow Center)

**Figure 10.**
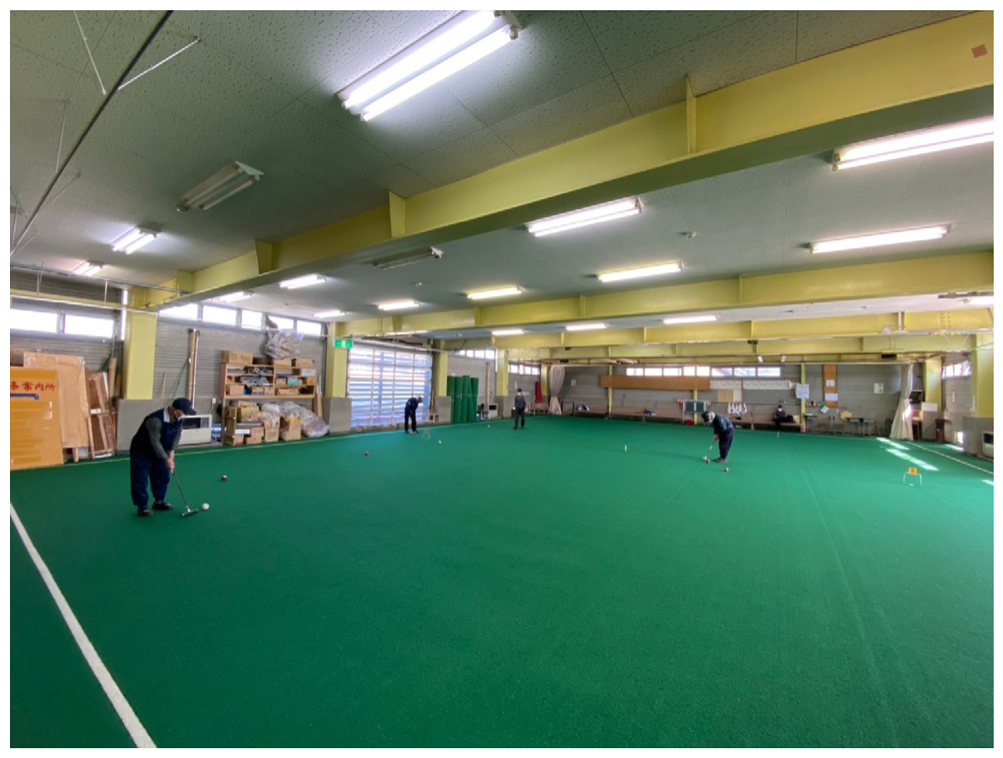
Tokamachi Snow Center in Winter 2020. Photo by Maharu Maeno.

**Figure 11.**
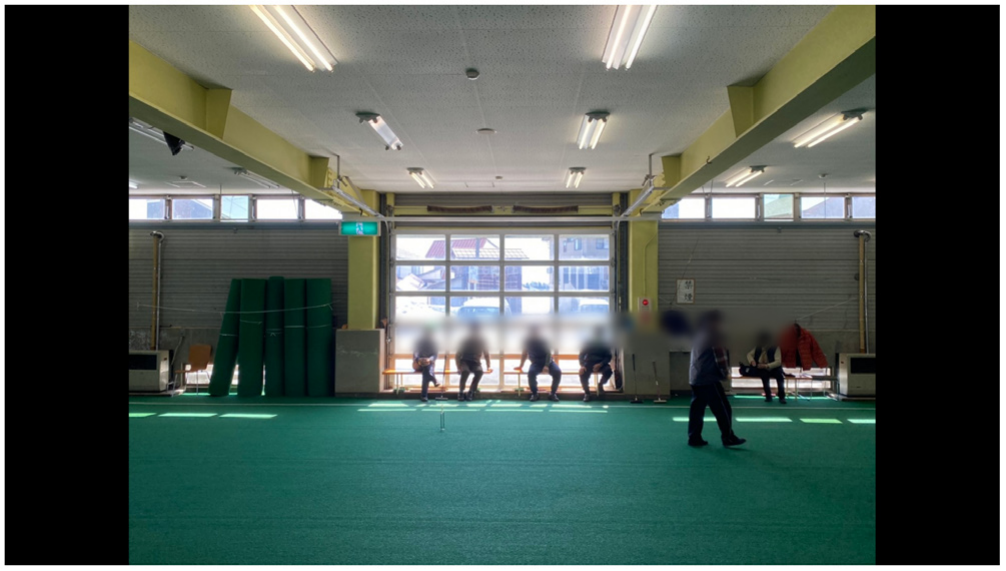
Tokamachi Snow Center in Winter 2020. Photo by Maharu Maeno. Figure 11 has been reproduced with permission from participant A, B, C, D, E F and G. Edits were made for de-identification purposes with permission from Maharu Maeno.

Our installation was housed in the Risetsu Shinsetsu Sougou Center, Tokamachi Snow Center (our translation), a former bathhouse surrounded by rice fields. In the winter, the two-floor building serves as an emergency shelter and a croquet ground ( Figure 10 and Figure 11). Our multimedia archive stood on the field’s artificial grass.

**Figure 12.**
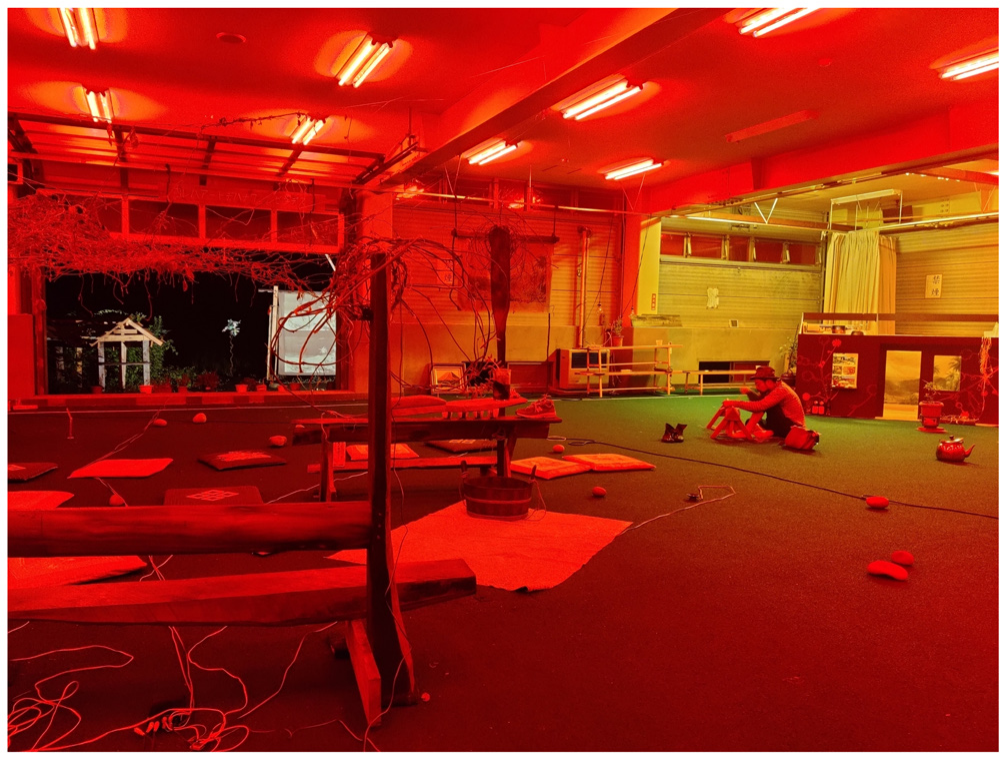
Threading kudzu vines (Pictured: J. Yamaguchi). At his back is the scaled-down version of El Arenero Yumita. Photo by Catalina Fernandez.

**Figure 13.**
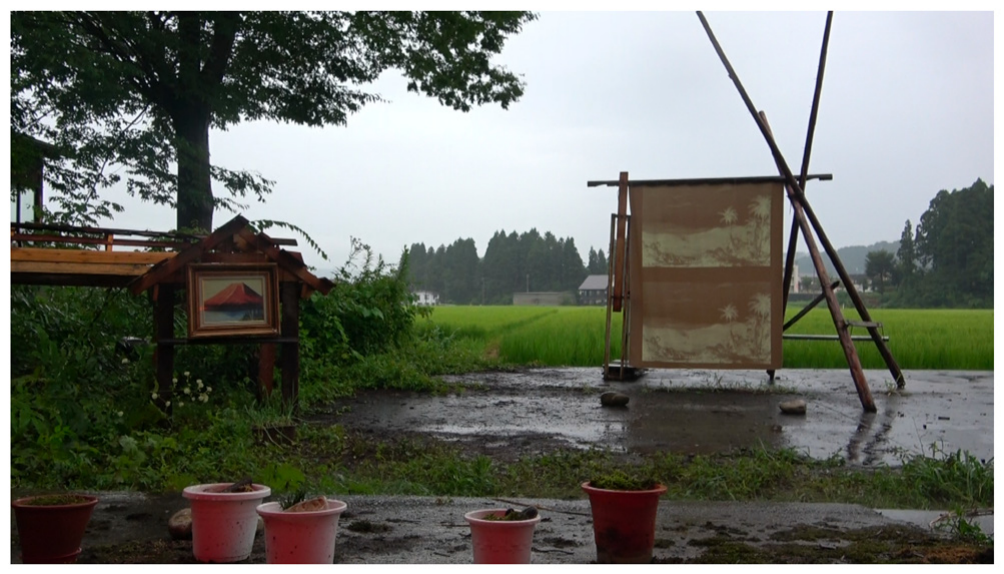
Outside, the scaled-down version of Yamaneko Guest House with a portrait of Mt. Fuji and a double reproduction of Frederic Church’s portrait of Mount Cotopaxi. Photo by Julie Lee. Figure 13 has been reproduced with permission from Julie Lee.

**Figure 14.**
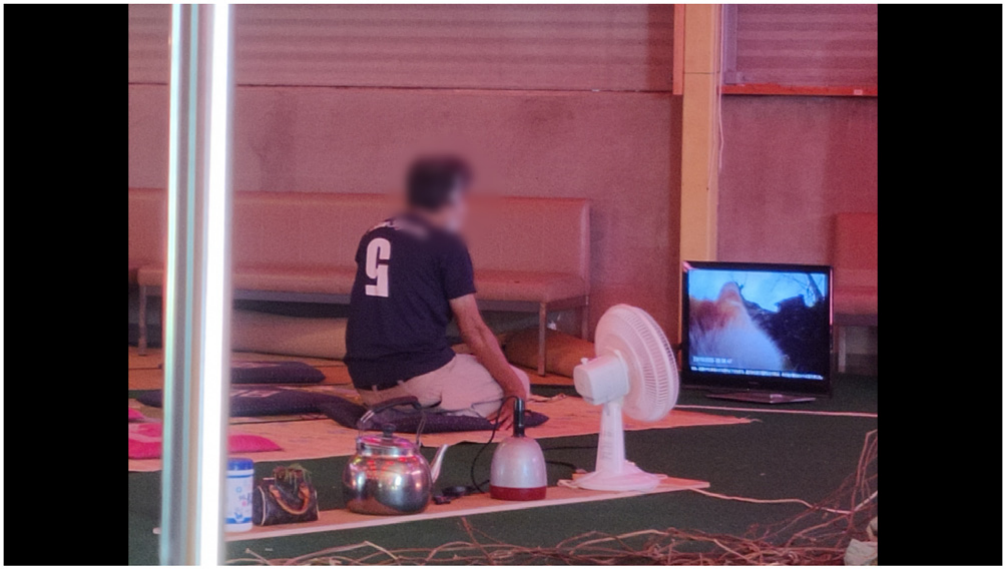
Detail of our installation *Clumsy-Seeming Mountains* in 2022. Photo by Catalina Fernandez. Figure 14 has been reproduced with permission from participant H. Edits were made for de-identification purposes with permission from Catalina Fernandez.

The installation emulated a sci-fi bathhouse. We installed red filters in the overhead lamps and windows and played a sci-fi soundtrack composed by Catalina using sounds from natural environments and electronic noise ( Figure 12). The whole experience made the cricket field seem slightly “off” to locals and visitors. The deep red of the interior gave the greens a particular intensity, a visual illusion similar to the water stage in Min Tanaka’s Butō performance, highlighting the presence of rice fields around our installation. ^12^ In the field, we placed TVs with our video diaries, documentation of the vine’s entanglements, three-dimensional kudzu-threaded mountains, dance scores, poems, and a model of El Arenero Yumita’s house. Outside, we installed a scaled-down version of Yamaneko’s Guest House and a double print of Frederic Church’s portrait of Mount Cotopaxi ( Figure 13). ^13^ Visitors were invited to explore the grounds barefoot, encounter the trembling mountains, and have a glimpse of our video and photo archives ( Figure 14).

By placing MaCA’s archives, we aimed to link the collective’s past to the rice fields and the mountains, a living archive just outside of the exhibition space. However, the presence of rice fields slowly transforms outside and humans harvest rice according to the seasonal cycle. Rice fields represent the time-space between nature as wild and as tame. The performance of rice fields enmeshes people in plant agencies. Even though rice fields have been heavily domesticated, they continue to exhibit behaviors that are not solely driven by human desires; rather, they also demonstrate their own performances. Indeed, visitors are aware of the performance of rice fields and watch three huge mountain objects, each about three to five meters in diameter, made by collecting and weaving local kudzu, hung in the space. Mountains made of kudzu in the building and the real mountains outside guided the visitors to in-between perspectives. Mountains were vessels of organisms and living archives in order that visitors walked around inside/outside of mountains, staying close to Earth beings. Thus, the choreography of the clumsy-seeming mountains reopens the discussion of ecological considerations and brings a sense of kinship.

**Figure 15.**
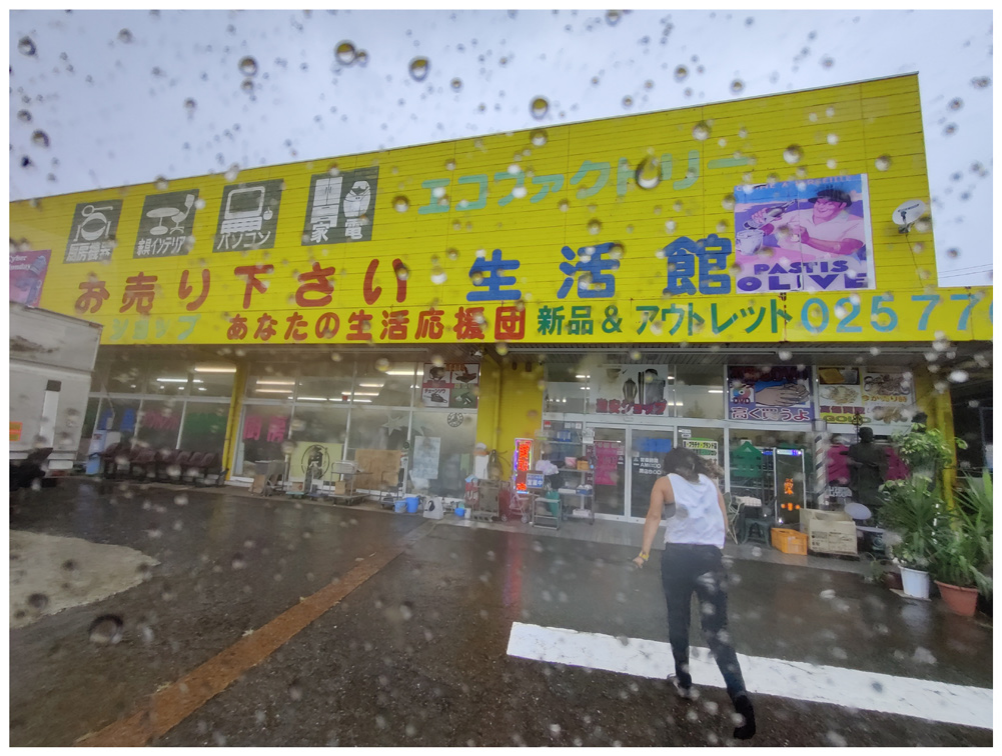
Collecting materials at Eco Factory in Muikamachi, 2022 (Pictured: A. Viteri Arturo). Photo by Catalina Fernandez.

Surprisingly, we struggled to find second-hand, recycled materials for the installation. The vast amount of e-waste produced in Japan is a serious concern for the Japanese environmental movement, ^14^ and the use of second-hand materials does not seem to be a common practice. We searched for used headphones, cables, and transmitters in the basement of a second-hand, recycled materials store in Akihabara, Tokyo. As plastic packaging has become an omnipresent part of our daily existence in Japan, we found it challenging to adhere to eco-friendly practices ( Figure 15).

## An emergent form of ecological performance: “Turn Off the House Lights,” an immersive performance

**Figure 16.**
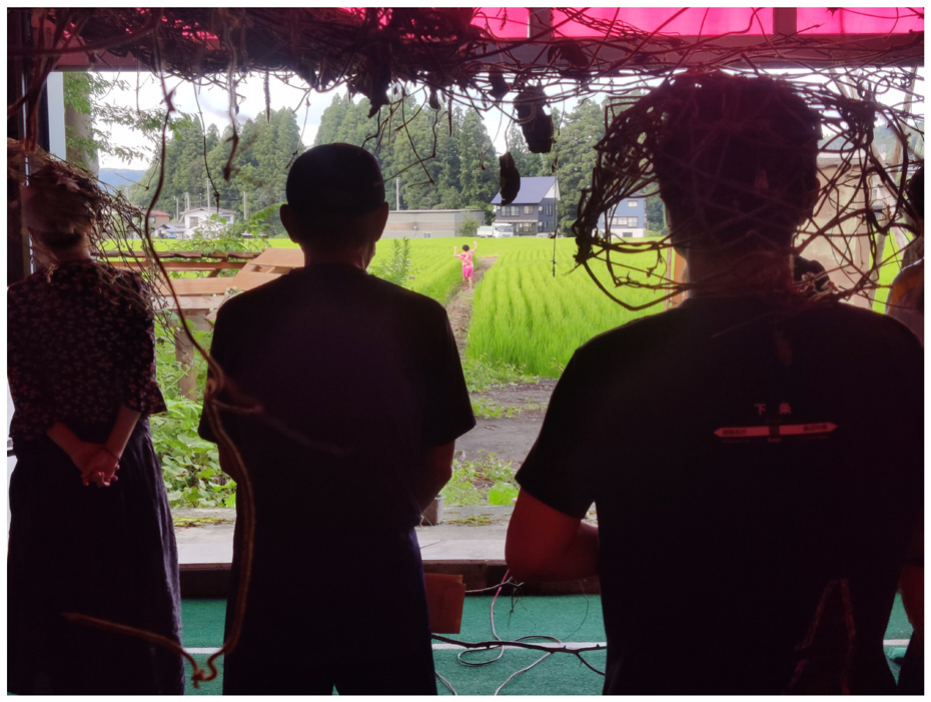
Rice field dance, “Turn Off the House Lights,” 2022 (Pictured: S. Yoshida). Photo by Catalina Fernandez.

During our time at Echigo-Tsumari Art Triennale 2022, we listened to rice farmers, local volunteers, and sightseers. For our contribution to the exhibition, we shared MaCA’s atmospheric performance “Turn Off the House Lights,” adapting the script to the region’s stories and integrating movements and gestures inspired by our interactions with the landscape and the people. “Turn Off the House Lights” transformed our embodied research into a tangible experience, immersing our guests in the sensuous realms of real, imagined, and imaginations-of-real landscapes ( Figure 16). ( Tamler, 2022)

**Figure 17.**
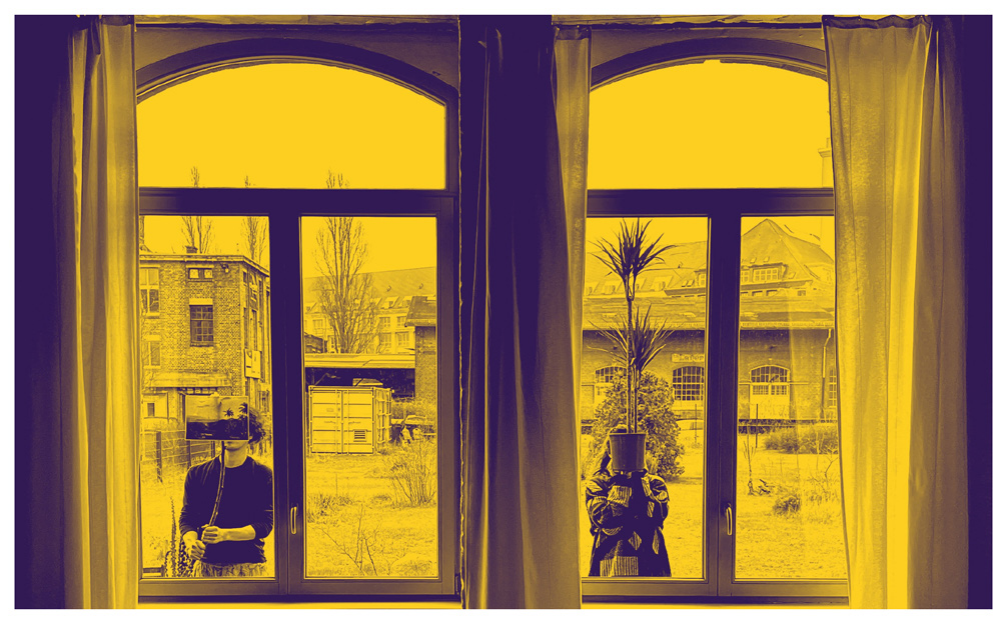
Instagram post, “Turn Off the House Lights” at Cordillera Berlin, 2022 (Pictured: S. Yoshida and A. Viteri Arturo). Photo by Maharu Maeno.

In the Berlin rendition of “Turn Off the House Lights,” we guided guests wearing wireless headphones from a dance studio to a horticultural garden, stewarding them through an imagined forest ( Figure 17). The outside community garden features several islands of flowers and plants cared for by neighbors. Together, we meandered – something like taking a walk while chatting on the phone with a loved one or sitting with a group of friends to listen to a thunderstorm. We convened around a bonfire to witness the sunset. Our narrations layered Tokyo’s urban views with Frederic Church’s portrayal of the Andean mountain chain. ^15^ Our script aimed to challenge colonial interpretations of the landscape and delved into the mountains’ spiritual lives. ( de la Cadena 2015, 25)

**Figure 18.**
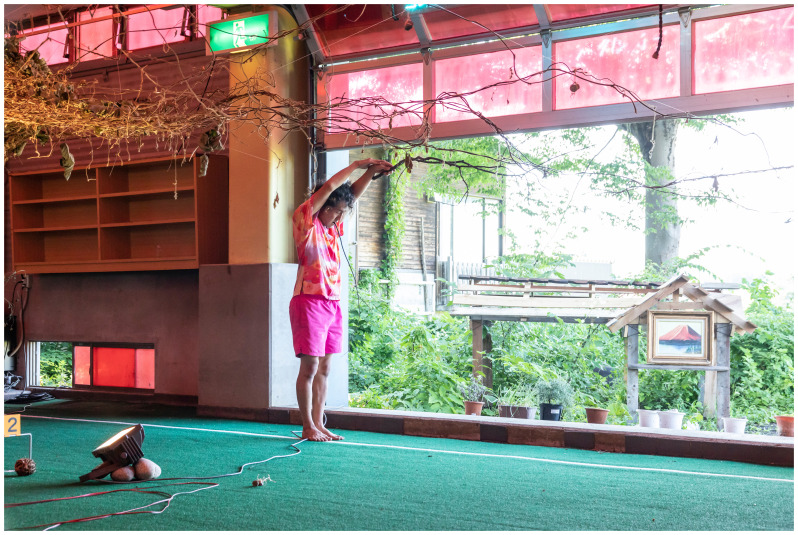
Cherry tree beside the river in “Turn Off the House Lights,” 2022 (Pictured: S. Yoshida). Photo by Osamu Nakamura. Figure 18 has been reproduced with permission from Osamu Nakamura.

Conversely, the Japanese version incorporated embodied renditions of our visits to Takaba mountain, gestures developed while handling kudzu, and stories from local rice field farmers. We wanted visitors to have an immersive experience that reflected the experience of viewing Mt. Fuji while submerged in a hot spring, part of Japan’s bathhouse culture. While Maharu and Alex told stories, Catalina mixed our soundscape live, Jun sewed kudzu fibers, and Shuntaro performed non-human choreographies – he became a spider in a bathhouse, a bird in a rice field, the pine tree in Church’s paintings, a cherry tree beside the river in Kotō-ku, a kudzu vine, and one stalk among the many in the rice fields. Emerging from the back of the rice fields and returning to them towards the end, he describes the experience as the following of disobedient movements, something in between the wildness of the mountains and the domesticated nature of the fields ( Figure 18):

I tried to draw every movement from the environment. I moved in response to the wind, the clouds, the weaving of the rice fields, followed mountain ridges, and channeled distant rainfalls. The more I was invested in their movement, the lighter my movements became. In the in-betweenness of domestic and wild, the body’s senses did not know which nature it was responding to. ^16^

In the performance of “Turn Off the House Lights,” the rhythms of the body and nature resonate through the sensory and perceptual (re)organization induced by other-than-human persons’ choreography. Dance scholar Tamara Ashley’s proposal for ecological choreography introduces and expands upon the notion of performances that are primarily linked to place. ( Ashley, 2012, 27) Going against this, dance and theatre writer Maša Radi Buh explains that the location is sequentially changed and updated in trans-geolocation choreography through the sound walk. She mentions the updated ecological choreography in the following:

Ecological choreography not only incites an ecological experience of awareness through theatrical content but offers an experience through the medium itself as well. The difference between the former and the latter is that one of them counts on the audience’s feelings while the other engages them with embodied ways of attending and perceiving.
  ( Buh, 2022, 47)

Maša proposes a geolocational multimedia soundwalk as ecological choreography. Such a performance engages the attendee through a physical attunement which is connected to two interpretations of listening, both connected to artistic practices aiming to aid the body in experiencing some type of ecological awareness. Participants listen to a set of audio tracks which correspond to certain locations along the route, with interconnected elements giving the sense of a unified event. She describes the connection between the three elements of space, time and sound, and the connection “exemplifies the principle of interconnectedness taken over from ecology as the change or fault in the execution of one of them significantly alters the performance itself, as well as the participant’s experience.” ( Buh, 2022, 47–48) Maša’s ecological experience of awareness indicates attentive walking and recalls the absence of the noises of civilization during the COVID-19 pandemic. In our performance in Berlin, we challenged the soundwalk, inviting gardens into an embodied practice. However, simply told, our performance at Echigo-Tsumari Art Triennale 2022 couldn’t avoid theatrical frameworks such as the division of space between stage and auditorium space because we couldn’t use remote headphones in Japan. ^17^ During the performance in Echigo-Tsumari, guests were located between live narrators (behind guests) and performer, and the fixed location prevented the changes and contingencies of participants moving the environmental structure.

## Conclusion

This essay has presented our collective research-creation and “Turn Off the House Lights,” a work of other-than-human choreography. The choreography navigated the border zone between human and non-human, which emphasized ecology territories inciting an ecological experience of awareness. Our ecological performance is not only a constant opening of new imaginative spaces by surrendering and reacting to the (in)visible and disordered gestures and irregular movements of living and inanimate living objects, but also a narrative choreographic practice that is intertwined with the global environment in each place and with each time. During site research, we were looking for entanglements, tuning to the territory’s ecologies. We actualized the script with the voices and atmosphere of each place, responding to our host environment. Therefore, in these living archival practices, the other-than-human persons’ choreography is effectively expressed in cross-disciplinary ways – visual art, music, lighting, dance, theatre, and text – while at the same time finding clues to respond to the future ecosystem through the back-and-forth of the incommensurable noises of the malfunction and disobedience of which Earth’s beings are composed.

**Figure 19.**
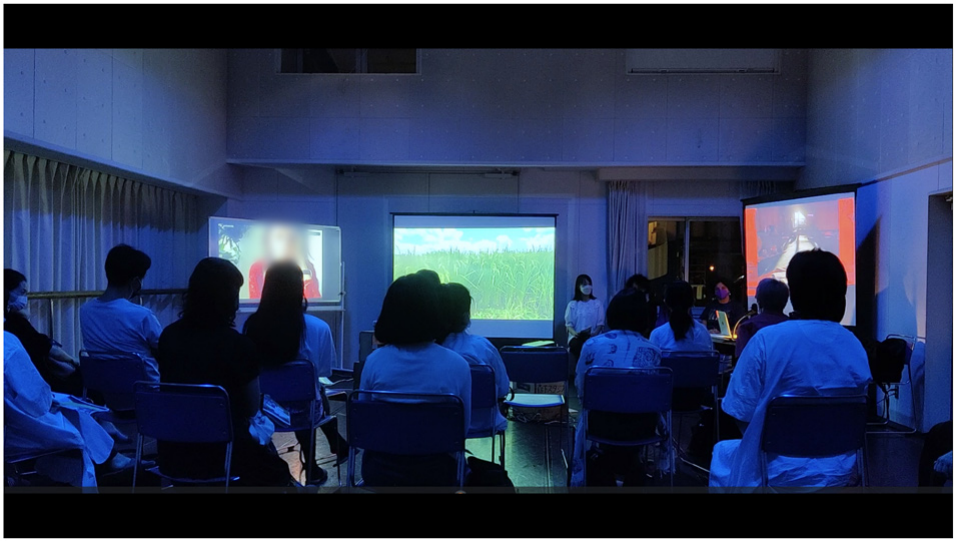
“広い島/HIROSHIMA NO TIENE NADA QUE ENVIDIARLE A PARIS,” hosted by The Saison Foundation, Tokyo, 2022 (Picured M. Maeno and C. Fernandez). Photo by Taro Inamura. Figure 19 has been reproduced with permission from Taro Inamura. Edits were made for de-identification purposes with permission from Taro Inamura.

Writer, translator, and interdisciplinary artist Cory Tamler interviewed us at Morishita Studio after the performance of “Turn Off the House Lights” at the Echigo-Tsumari Triennale ( Figure 19). She wrote about our work:

[The] research and creation process is one of shared archiving, a kind of living accumulation – analogous, in my mind, to the simultaneous growth and decomposition in a garden. The collective (de)composes their archive through the exchange of gardening techniques and a “practice of encounter” (in their words) with non-humans, but also through their varied artistic practices, from sound and visual art to dance.
  ( Tamler, 2022)

Our living archival practices are comprised of the transmission and performance of other-than-human persons’ lives and forms.

From digital residency and site research to installation and performance, our living archives accumulate, on the one hand, the conceptual malfunction that spills over from material exploration. The malfunctions that appear during the creative process address phenomena beyond the human and open the way for an escape from the anthropocentric perspective. On the other hand, a feeling of substantive disobedience spills over from transcendental exploration. The disobedient movement of other species dissolves the dichotomy of human and non-human, and changes to the tuning of their rhythm in an intentional break from the dramaturgical framework. Our senses shifted back and forth in the encounters with other-than-human persons’ choreography, underlining the way other-than-human approaches reflect the complex relationship between humankind and the natural world. However, living archival practice in a collective life can contribute to concern and care for both the Earth and the disobedient movement of its living beings. This practice helps sustain the collective lives of other-than-human persons and their ecosystems.

At the time of writing, our artistic practice only incites the awareness of environmental awareness. Furthermore, considering the Earth’s rapid deterioration over a decade, it is significant that we consider how artistic works represent ecological choreography. Despite the development of theories of the Anthropocene and posthumanism, these have primarily been applied to science and artwork rather than interdisciplinary research. Thus, we argue for a more radical development of embodied practices and interactions between human and non-human environments to elicit a new investigation of the relationship between the human and the non-human that may be experienced by audiences. Most importantly, cross-disciplinary perspectives should transform collaboration in the collective through encounters with other-than-human persons, opening the way to further living archival practices.

## Ethics and consent

Ethical approval and consent were not required. The authors confirm that consent to publish the name of Luz Marina Giraldo. Any identifiable information has been used with explicit permission for publication. Regarding the consent to publish identifiable details, written informed consent was obtained via her family member because she has passed away.

## Data Availability

No data are associated with this article.
