# Living archival practice and the choreographical navigations: Encounters and approaches with other-than-human persons

**DOI:** 10.12688/openreseurope.17447.2

**Published:** 2025-11-29

**Authors:** Shuntaro Yoshida, Alex Viteri Arturo, Catalina Fernandez, Maharu Maeno, Jun Yamaguchi

**Affiliations:** 1 Waseda University, Shinjuku, Tokyo, Japan; 2 Berlin University of the Arts, Berlin, Berlin, Germany; 3 CUNY The Graduate Center, New York, New York, USA; 4 Independent researcher, Berlin, Germany; 5 Weißensee Kunsthochschule Berlin, Berlin, Berlin, Germany; 6 Tokyo City University, Setagaya, Tokyo, Japan; 7 Musashino Art University, Kodaira-shi, 1588557, Japan

**Keywords:** Other-than-human persons, malfunction, disobedient movement, choreography

## Abstract

This article delves into the collaborative work of the interspecies dance collective, Mapped to the Closest Address (MaCA), focusing on MaCA’s living archival practice and exploration of choreography with other-than-human persons. Through encounters with various species and environments, MaCA seeks to shift anthropocentric perspectives, interrogate its orientation towards modernity and coloniality, and question its understanding/administration/entanglement/devotion of, with, and to nature. The collective’s journey, from a digital residency during the COVID-19 pandemic to site research, installations, and performance at the Echigo-Tsumari Art Triennale 2022, is documented and analyzed. The collective’s collaborative process involves relinquishing control to allow for the emergence of disobedient movements and the exploration of choreography from the perspective of other-than-human perspectives. This practice includes encounters with kudzu vines and mountains, weaving their movements and patterns into performances and installations. The authors discusses the immersive performance *Turn Off the House Lights*, in which MaCA integrates stories from local communities with gestures inspired by the landscape. Through the collective’s living archival practice, MaCA aims to transmit a collective memory of intra-actions among organisms and environments. and highlight the intra-connectedness of humans and the other creatures of the Earth. The article reflects on the significance of choreography beyond human-centric notions, emphasizing the emergent forms of ecological performance and the dissolution of boundaries between human and non-human realms. Drawing on interdisciplinary perspectives including dance, visual art, and theatre, MaCA’s work exemplifies an intra-disciplinary approach to expressing the choreography of other-than-human persons. This approach not only presents audiences with immersive experiences but also responds to the future ecosystem through artistic exploration. Ultimately, MaCA’s living archival practices contribute to awareness of the collective lives of other-than-human persons and offer insights into navigating the collective’s enmeshment with the natural world.

Human beings are part of nature.

( Echigo-Tsumari Art Field, n.d.)

## Introduction

From 2019 to 2022, Mapped to the Closest Address (MaCA) was an interspecies dance collective composed of four human animals (Colombian performance maker Alex Viteri Arturo, Colombian sound/light designer Catalina Fernandez, Japanese visual and performance artist Maharu Maeno, and Japanese dancer/choreographer Shuntaro Yoshida) and a cat, Violeta, who participated as a non-human videographer and relational being. Through choreographic practices, the humans in the group sought to interrogate MaCA’s orientation towards modernity and coloniality, question its understanding/administration/entanglement/devotion of, with, and to nature, and shift their anthropocentric perspectives. The concept of the Anthropocene highlights that the human-induced destruction of nature began in earnest as societies industrialized, and the human-centered view of nature accompanying the term “Anthropocene” holds the key to retreating from modernity/globalization. As historian Dipesh Chakrabarti indicates, this encourages a historical dialogue between the non-human and the human species living on the planet. However, when discussing the disrupted collective environment, it is essential to note that the terms “Anthropocene,” “human species,” and “nature” are tied to human agency. While the Anthropocene started in the industrial societies of the 19th century, environmental historian Jason W. Moore points out that the pre-industrial 16th century was equally important—the global system through which natural resources are bought and sold at artificially low prices was dramatically expanded by Western colonialism starting in the Age of Exploration. Humans view nature as a resource and render it into capital, creating an imbalance between humans and nature. Cultural critic and feminist Donna J. Haraway and geographer Kathryn Yusoff offer a different critique of the concept of the Anthropocene. ^1^ Furthermore, theatre and performance studies scholar Una Chaudhuri shifts the focus from the representation of animals to their performance, referring to the emergence of reciprocal animality—a dynamic interrelation between humans and animals ( Chaudhuri, 2017, 31–48). In this vein, MaCA engaged diverse human and non-human players to consider on other-than-human persons’ performance as the mutual constitution of entangled agencies and landscape.

**Figure 1.**
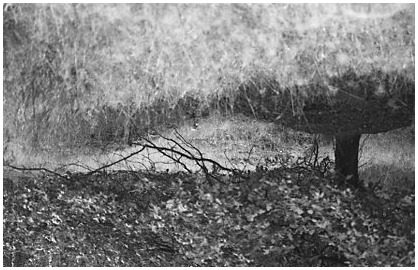
Mushroom field surrounded by forest at El Arenero Yumita. 35mm black-and-white film. Photo by Alex Viteri Arturo.

Thus, MaCA’s standpoint shifted from the notion of “the Anthropocene” and “more-than-human” associated with posthumanism to “Earth beings,” which approaches other species’ performance collaboration from a more symbiotic or altruistic perspective. Collectively, MaCA sowed practices to enter into contact with other-than-human persons. ^2^ MaCA used various devices to record its encounters and crafted multimedia social spaces to share its archive ( Figure 1). The following discussions of its collaboration explore the history of its encounters and research-creation.

MaCA participated in the Echigo-Tsumari Art Triennale (ETAT) in 2022. Before the opening of the festival, the collective explored three nearby mountains—Akiba, Gongen, and Takaba mountains. During these explorations, they encountered and collected kudzu vines, which shifted the collective perspective on the plant. Initially seen as an invasive species, kudzu revealed its intricate connections to the local ecosystem through its weaving patterns and growth behavior. Inspired by these observations, MaCA choreographed movements reflecting this entanglement and integrated kudzu’s weaving patterns into the *Clumsy-Seeming Mountains* installation. This choreographic score extended non-human choreographies, knitting together an understanding of how plants live and move on the land, embodying their intra-connected rhythms and lifeways.

This article considers MaCA’s research-creation, from its recollections of a digital residency in 2020 during the COVID-19 pandemic to site research, installation, and performance at the ETAT 2022 in Niigata. The discussion follows a digital residency conducted between Tokyo (Japan) and Frankfurt an der Oder (Germany) in 2020, the performance *Turn Off the House Lights* in Berlin in 2022 (further discussed in the conclusion), and site research in Tōkamachi City, Clumsy-Seeming Mountains and the performance *Turn Off the House Lights* in Tōkamachi City, Niigata, in 2022, and “広い島/HIROSHIMA NO TIENE NADA QUE ENVIDIARLE A PARIS,” an immersive talk at Morishita studio in Tokyo ( Table 1). The collective archived the movement of other species and collectively translated the awareness of local ecosystems within planetary and seasonal processes into its choreography. Artist Carolina Caycedo calls these “geo-choreographies.” This term clarifies “how a seemingly mundane every gesture [...] can be given agency through language. The term geo-choreography is charged with political power that can be put to use when the river is threatened. The deeper the connection with the locality, the stronger the agency of the geo-choreography” ( Ostendorf-Rodriguez, 2023, 34–35). Here, the choreography means the interactions of our bodies within that environment that hold knowledge, power and influence. Meanwhile, dance scholar and dramaturge Moritz Frischkorn speaks of “more-than-human choreography,” positing that dance or movement practice is not solely a human activity but a collaborative and relational endeavor involving various elements of the environment. This term avoids both the techno-romance implied by “post-human” and the care practice associated with “more-than-human” ( Frischkorn, 2023, 19–21). MaCA speaks of “other-than-human choreography” as an element of the intra-connectedness and intra-disciplinary perspectives united under the concept of *intra* ( Barad, 2007), emphasizing the ethos and dynamics of the more-than-human. In other words, through the accumulation of living archives, MaCA’s collective memory of intra-actions among organisms and environment navigates the other-than-human persons’ choreography in the context of confused the notions of the “wild” and “domestic” notions in creative practice. By considering non-human agents within the expanded history of choreography, we can begin to situate the choreographic object itself as non-human. For instance, dance historian and dramaturgist Anna Leon comments on a choreographed tree in choreographer William Forsyth’s *Groningen Project*. She observes that “people control nature, and choreographers define the movement of bodies – in an expansion of choreography, the choreographer controls nature and defines the forms that it will embody” ( Leon, 2022, 191). However, the collective theorizes an other-than-human choreography that challenges Western approaches to the non-human’s non-danceable movement and the more-than-human’s non-moving movement based on Western notions. A key point is the cross-cultural approach that offers the clumsy-seemingness which deconstructs a central point of view and proposes more symbiotic and altruistic worldviews and approaches.

The detailed examination of these perspectives in *Turn Off the House Lights* aligned with emergent ecological performances. This sound-dance performance was held at the ETAT in 2022. Its multi-species creation process, involving other species, has focused on the concept of the “intra-connectedness” of humans and non-humans. However, in this article, MaCA turns its attention to the approaches to and significance of the other-than-human choreography.

**Table 1.** List of MaCA’s research-creation, from a digital residency in 2020 to site research, installation, performance at the ETAT 2022 and an immersive talk at Morishita Studio.

*Name*	*Date*	*Place*	*Items*
Digital Residency *Open * *Forest Launch*	October 15–November 2, 2020	Morishita studio in Kōtō-ku (Tokyo, Japan) and El Arenero Yumita (Frankfurt an der Oder, Germany) Coordinates: 35°41'14.0"N 139°48'03.4"E (Morishita Studio) 52°17'49.2"N 14°28'32.2"E （Arenero Yumita）	Photo documentation Spy camera Two fax machines Gardens Video diary
*Turn Off the House Lights*, an immersive performance	March 4, 2022; May 21, 2022	CORDILLERA Raum für Körper und Utopien (Berlin, Germany) Coordinates: 52°27'30.2"N 13°31'40.9"E	Cotopaxi mountain pictures by Frederic Church (large and small sizes) Garden Microphones Props from garden (dead leaves, moss, trees)
Site Research at ETAT 2022	July 12–July 31, 2022	Three mountains (Akiba, Gongen, and Takaba) in Tōkamachi City (Niigata, Japan) Coordinates: 37°06'03.9"N 138°45'49.4"E (Akiba) 37°09'00.3"N 138°41'40.7"E (Gongen) 37°15'01.0"N 138°49'42.3"E (Takaba)	Kudzu *Satoyama* scores (Akiba, Gongen, and Takaba mountains)
Installation: * We like to * *watch the clumsy-seeming * *mountains* Immersive *p*erformance: *Turn Off the House Lights*	August12– November 5, 2022 Presentation: August 17–19 and September 10–11, 2022	Risetsu Shinsetsu Sōgō Center, Sanjo area, in Tōkamachi City (Niigata, Japan) Coordinates: 37°11'53.7"N 138°46'34.2"E	**Inside:** Artificial pine tree Cushions Indoor croquet lawn and equipment (mallets, balls, and goals) Fluorescent tubes with red covers *El Arenero Yumita* (installation; inside: photo documentation and objects from research) Kudzu-threaded mountains (head-worn type) Mount Fuji pictures Plants Print of Violeta (large scale) Sounds mixed from natural environments and electronic noises Stones Three *satoyama* dance scores (Akiba, Gongen, and Takaba Three huge kudzu-threaded mountains with three sound speakers Three monitors (videos: video diary, documentation of kudzu fermentation, and site research) Wooden benches made of wood reclaimed from Yamaneko Guest House, with secondhand headphones and devices **Outside:** Double print of Mount Cotopaxi by Frederic Church (large scale) with wooden pedestal Installation of Yamaneko’s Guest House, made of wood reclaimed from the original Rice field **Performance:** Fireworks Gloves Lighting and tape lights
“広い島/HIROSHIMA NO TIENE NADA QUE ENVIDIARLE A PARIS,” an immersive talk	September 12, 2022	Morishita studio (Tokyo, Japan) Coordinates: 35°41'14.0"N 139°48'03.4"E	Fluorescent tubes with blue covers Map of Kōtō-ku’s gardens Three projectors (two videos of Mount Fuji research and live screen for guest (Cory Tamler))

To explore the collective’s living archival practice, the authors look at how movements emerging from relationships with non-human beings have been practiced. The idea of attunement to nature took on new developments in the US in the 1960s through the work of choreographer and dancer Anna Halprin. Focusing on an awareness of the environment, Halprin explored the relationship between the human body and the natural environment through bodily awareness of her own movements and how they responded to the surroundings. Meanwhile, her husband, architect Lawrence Halprin, designed gardens for visitors and for dance. Both of them created scores (instructional frameworks) in which they pursued the relationship with nature, thereby forming a mode of interconnectedness with the environment through dance. Underlying their exploration through scores was Lawrence Halprin’s concept of the “RSVP Cycles.” As both an architect and Anna Halprin’s partner, Lawrence conceptualized the creative process as a cycle consisting of four elements: Resources, Scores, Valuaction (value assessment), and Performance. He presented this as a flexible framework in which one can move back and forth among the elements, engaging in iterative feedback. In particular, “Scores” were positioned as guidelines for engaging both sensorially and analytically with the “conditions of the site,” which included interactions with the environment and with others. By employing scores, Lawrence aimed to reveal “hidden obstacles”—barriers that, once made visible and recognized as hindrances, could be addressed through the RSVP Cycles ( Halprin, 1970, 3). Such “obstacles” constitute the very nature of a living archival practice, and for the authors, they signify the other-than-human choreography. The “entanglements,” “frustrations,” and “misunderstandings” that often appear in site-based practice are, within the RSVP framework, not things to be overcome, but rather catalysts for creativity. In other words, the detours whereby an agent recalibrates relations with the external environment and responds resonantly via the score correspond precisely to the existence of movement in malfunction within ecological choreography.

In contrast, the authors also draw on dance and theatre writer Maša Radi Buh’s notion of ecological choreography, which foregrounds not only the ecological experience of awareness but also the imagination of a future ecosystem—for instance, envisioning a post-apocalyptic time without humans through a geolocational sound walk that she mentioned. Furthermore, the choreography values “an (utopian) idea of a blurring of the distinction between (the value of) human and non-human creatures appears”( Buh, 2022, 48). Discourses on choreography, when mediated through “ecological awareness,” reopen the questions around how choreography by other-than-human beings works in practice. Today, thinking about dance and choreography can no longer be confined within the frameworks of human perception, intention, and technology. Rather, choreography can be understood as a process of response to local environments, plants and animals, minerals, sounds, gravity, and even invisible existences—“alien” others. This process is often triggered by deviations known as “malfunctions” or “disobedience.” Such deviations do not simply disturb an anthropocentric order; they themselves open up new choreographic possibilities. As Nina Lykke points out in her posthuman phenomenology and poetic practice, it is crucial to avoid the double trap of either anthropomorphizing alien others or distancing them as wholly incomprehensible beings ( Lykke, 2024, 80–84). From this critical perspective, we must assess how “movement in malfunction” can demonstrate an ethical sensitivity within creative processes

This article examines its ecological choreography strategies via malfunction and disobedient movement, revealing how such movements expose the dissolution of the border between human and non-human. Movement in malfunction designates to a resonant movement that emerges through the intertwining of human movements derived from contact with the environment or other-than-human persons and the movements generated by non-human choreographies. It attempts to shift controllable movements into disobedient ones. Relearning “planetary kinship” thus becomes an essential task in choreographic practice. This entails moving away from modern thought that privileges the human, rediscovering symbiotic connections between humans and other beings—plants, animals, minerals, weather, microorganisms—and bodily reweaving these ethical resonances. Dance, in this sense, functions as a medium for attempting, experimenting with, and documenting such relationalities at the level of the body. Within this framework, movement in malfunction is positioned as both a deviation and a creative circuit toward planetary resonance.

Furthermore, from the perspective of more-than-human choreography, feminist theorist and physicist Karen Barad’s notion of intra-action offers an important insight. Ecology here is not only about relations between living beings, but also about entanglements of matter itself, in which all agencies—human and non-human, animate and inanimate—emerge through their intra-actions. In MaCA’s living archival practice, this extends beyond relationships with nature to include the entanglements of materialities themselves, pointing to an intra-connectedness that we can glimpse within artistic practice. In this respect, this paper seeks to understand how MaCA perceives and receives such intra-connectedness, and how it might open the conditions for disobedient movement of other-than-human persons.

Specifically, MaCA aims to act out the transmission of its collective memory of landscape by encountering other-than-human persons. MaCA’s creation then shifts to tuning to local ecologies, and the collective encounters the notion of “disobedience” through the choreography of other-than-human persons. To illuminate the experience of heightened ecological awareness generated by disobedient movement, MaCA speculates on the choreographic navigations.

## Malfunctioning video and non-human choreography: *Open Forest Launch*, a digital residency (Frankfurt an der Oder/Kōtō-ku, Tokyo)

MaCA tended two vegetable gardens: a family’s backyard garden in Hyōgo (Maeno and Yoshida) and a *Schrebergarten* in Frankfurt an der Oder (Fernandez, Arturo, and Violeta). MaCA named the latter “El Arenero Yumita” as a tribute to American scholar of Chicana feminism Gloria Anzaldúa and Fernandez’s mother Luz Marina Giraldo “Yumita.” The garden represented a wishful naming for its queer family sanctuary and place to encounter and learn from other-than-human beings. ^3^ Through MaCA’s horticultural practice and research, the collective strived to cultivate a heightened awareness of planetary and seasonal processes and translated them into its notion of choreography. ^4^

**Figure 2.**
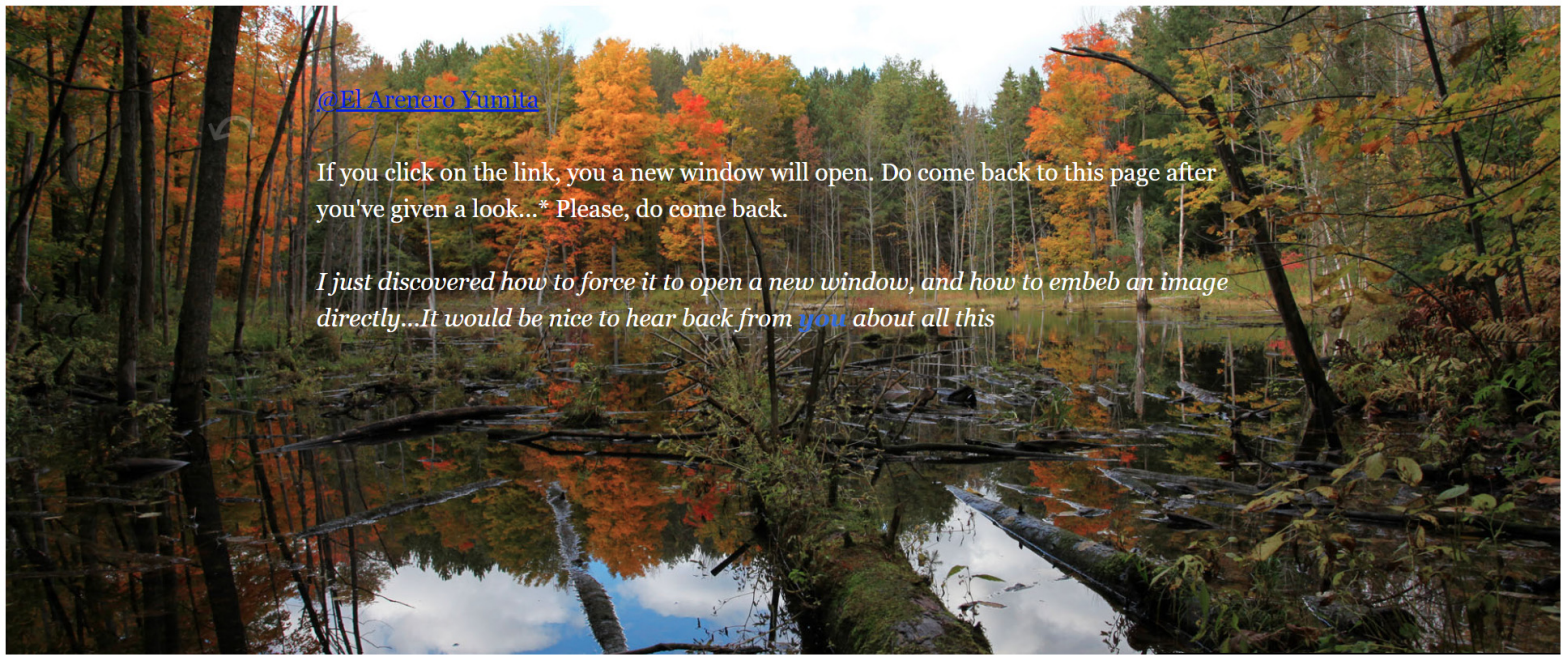
Screenshot of *Open Forest Launch* in progress. Photo by Catalina Fernandez.

Unable to travel during the COVID-19 pandemic, MaCA worked remotely, devising a digital residency to exchange the sensory experiences of its respective locations. Hosted by the Saison Foundation, MaCA investigated various ways to relate to non-human beings. ^5^ Its practices combined readings on non-human personhood with gardening. Non-human personhood denotes an active, sui generis mode of existence—neither a metaphor for the human nor something “less than” human—invoked to foreground the relationship between humans and non-humans. This led to “Open Forest Launch,” a collection of videos documenting its exchanges uploaded to a digital world still in progress ( Figure 2) ( Mapped to the Closest Address, 2020a).

**Figure 3.**
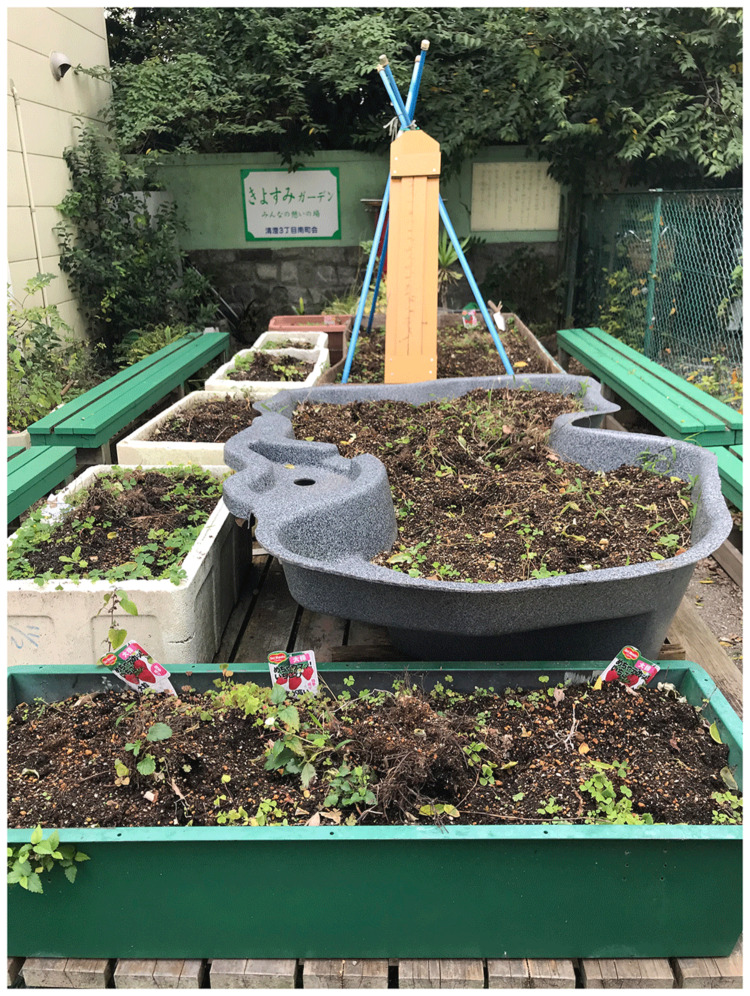
Kiyosumi community garden in Kōtō-ku. Photo by Shuntaro Yoshida.

**Figure 4.**
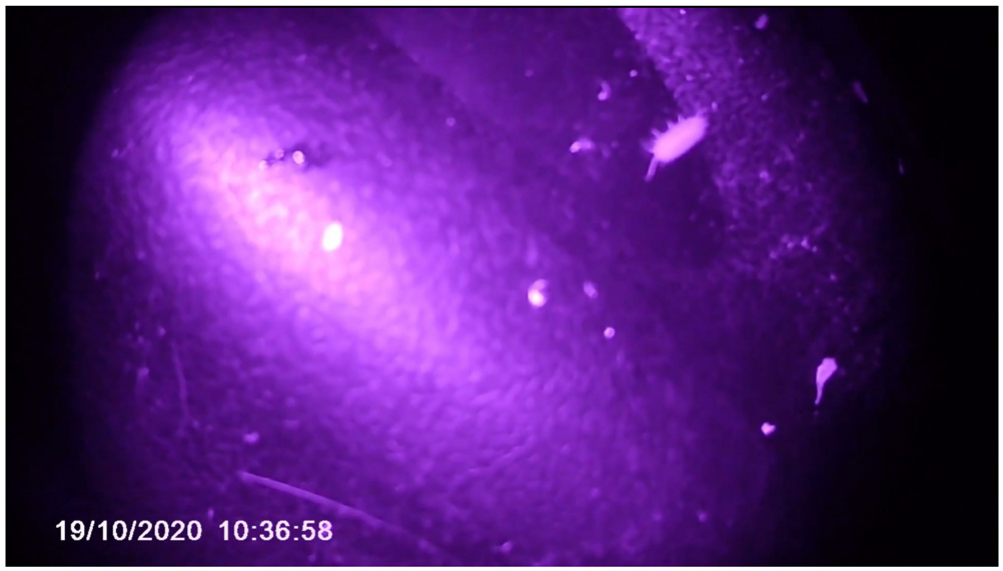
Scene from *Open Forest Launch_Video Diaries*. Captured by Violeta and edited by Catalina Fernandez.

**Figure 5.**
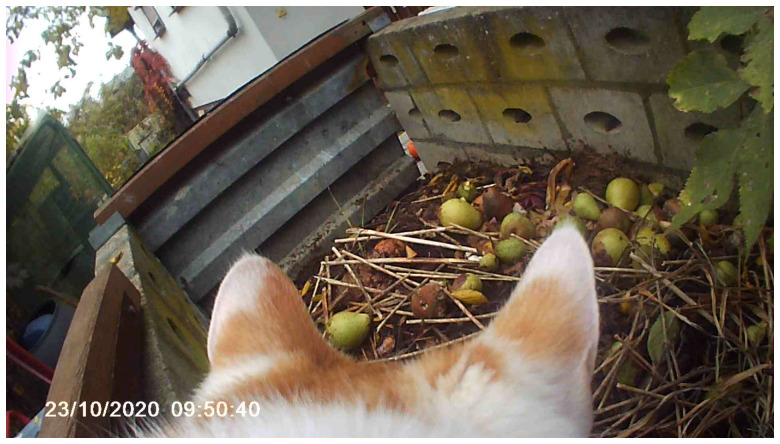
Scene from *Open Forest Launch_Video Diaries*. Captured by Violeta and edited by Catalina Fernandez.

In the video diaries, the collective witnesses the slow growth of vegetables, join the Kiyosumi community garden in Tokyo ( Figure 3 and Figure 4), and gazes into the depths of the Hellenesee, a sinking lake on the Germany-Poland border ( Mapped to the Closest Address, 2020b). MaCA sought to draw attention to diverse human and non-human players to dislocate its anthropocentric standpoint and explore from other points of view the many worlds within gardens, forests, and lakes. A cat, Violeta joined the collective as a non-human videographer, and her material became a fundamental part of the work. She made several field trips with a miniature spy camera attached to her back. Violeta’s footage served as one of the living archives that revealed and documented how she moved. Similarly, MaCA reimagined her life and world through her actions, such as following her gaze and climbing up trees, and communicating with crows alongside her ( Figure 5). In her video, MaCA noticed the impossibility of translating concepts between the humans and the non-humans, so the collective decided to cohabit with non-human movement.

The camera captured a view from a much lower position than a human’s camera would be placed and produced seemingly chaotic images. MaCA was exploring with the camera, which was bound by her pace and her four-legged locomotion. It is in this context that malfunction appears, as Massumi argues, within a “zone of indiscernibility,” where the conceptual distinction between the “domesticated” and the “wild” becomes blurred. ( Massumi, 2014, 8–9). Accepting the malfunction of the video was also a way of relinquishing mastery over the final art product. As the camera had been given to Violeta, human choreography committed to her and there was no way MaCA could control her movement: where she went, how long she decided to stay in a spot, and how fast she was. The collective relinquished the mastery over the video capture to Violeta. The video diary, including her perspectives, was also an attempt to take its thinking past human phenomena to a place beyond the human. Thus, the footage served as a portal that allowed MaCA to escape, although briefly, from its human perspective.

## Kudzu and mountains as other-than-human choreography: Clumsy-seeming mountains and site research (Tokamachi)

**Figure 6.**
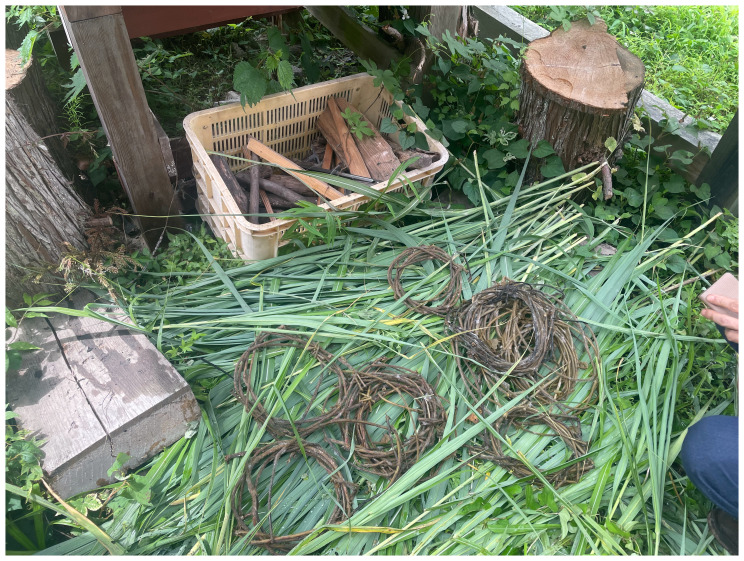
Maharu Maeno and Jun Yamaguchi cutting kudzu. Photo by Shuntaro Yoshida.

**Figure 7.**
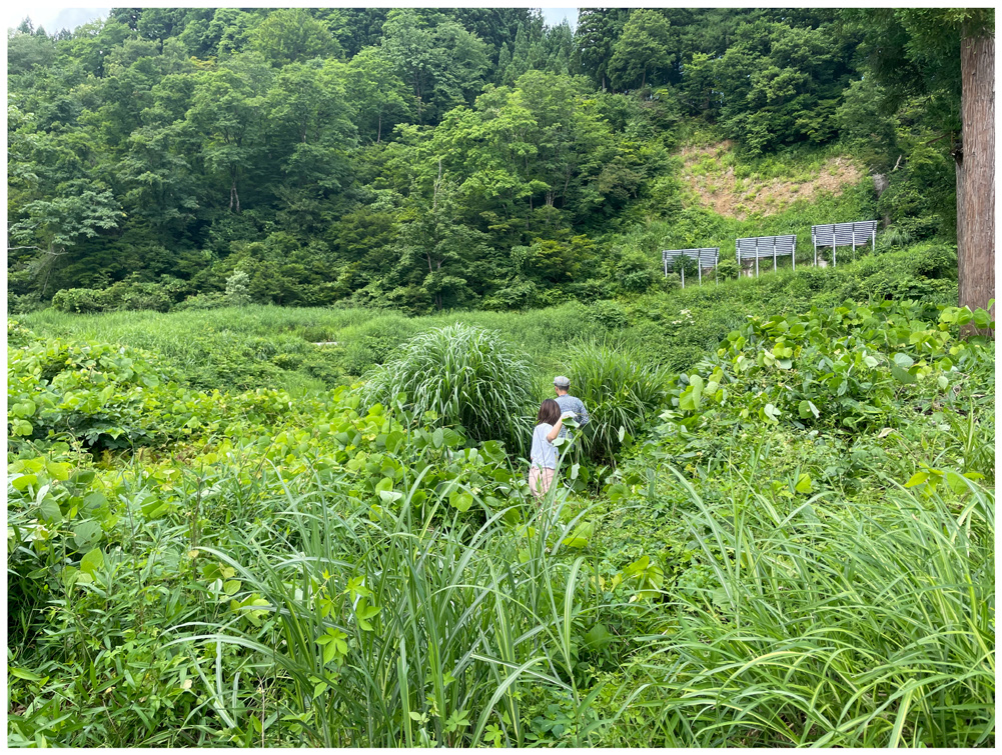
Kudzu fermentation (Pictured: J. Yamaguchi and M. Maeno). Photo by Shuntaro Yoshida.

MaCA received a second invitation, this time to participate in the ETAT. ^6^ In accordance with the Triennale’s guiding concept and ecological stance, MaCA managed to use 90% found natural objects in its performance and only the sound and lighting equipment introduced manufactured plastic goods. The collective traveled several times to the Shimojo area of the city of Tōkamachi, visited its assigned space, and planned for field research. The city of Tōkamachi is surrounded by mountains, which are called *satoyama*. ^7^ Prior to the opening of the festival, Maeno, Yoshida, and architect Jun Yamaguchi ^8^ stayed at Yamaneko Guest House to explore the three nearby mountains: Akiba, Gongen, and Takaba. They encountered and collected kudzu vines from up in the hills around the guest house ( Figure 6). While craftsmen of the ancient Jōmon periods (c. 14000 to 300 BCE) utilized kudzu for fibers and cloth, few such techniques remain. Today, the vine is deemed invasive and categorized as a weed. However, after engaging in processes like boiling, retting ( Figure 7), and river-washing to gauge its durability, the collective shifted its perspective on kudzu. MaCA recognized its intricate connection with kudzu and choreographed movements reflecting this entanglement. The collective learned from kudzu’s weaving patterns, integrating them into their mountain portraits for the installation. Portraits of Mount Fuji and Mount Cotopaxi emphasize that mountains have not only been perceived as natural landscapes but also represented, idealized, and inscribed with meaning through human-centered perspectives. In contrast, MaCA followed the patterns as if dancing, by handweaving the dried vines. The choreographic score comprised, in Yamaguchi’s words, “covering,” “spreading in search of the sun,” and “intertwining.” These extended non-human choreographies were knitted again to how plants live and move on the land, and the collective noticed their life compositions. The human role here was not to author choreography for the plant, but to attend to, inherit, and translate existing vegetal patterns into artistic practice. In MaCA’s case, choreography meant handweaving dried leaves while attuning to kudzu’s rhythms. In this sense, choreography emerges through entanglement: the plant offers its patterns, and human bodies respond, reframe, and render them perceptible as art. Thus, the collective stayed close to kudzu in terms of its embodied knowledge as inheritors of their movements.

While contemplating the mode of existence of kudzu and the functional workings of these life forms, MaCA explored the choreography of the intelligence of plants through the practice of listening to their rhythms and movements. Their leaf tips were growing very vigorously in the summer. Listening to and cohabiting with their movements, the collective relearned the guidance of kudzu. Becoming attentive to kudzu also opens up an intra-action with the mountains, the “Earth beings” which allow MaCA to engage in a sensory dialogue with organic matter. ( de la Cadena, 2015, 105). ^9^ This attentiveness gradually expanded beyond the plants themselves, attuning the collective to the geological and spiritual agencies shaping the surrounding mountains.

To this day, kudzu cloth continues to be crafted in Kakegawa. The mountains transmitted knowledge to MaCA, and the collective was tuning in to their rhythms rather than the other way around. While researching the Akiba 秋葉, Gongen 権現, and Takaba 高場 mountains, which surround the exhibition site, the collective encountered intra-connectedness between humans and non-humans. At the summit of Akiba mountain stands a shrine dedicated to Akiha Sanjakubō 秋葉三尺坊 or Akiha Gongen 秋葉権現, who is a manifestation of the Buddha as a Shintō god of fire protection appearing as a long-nosed Japanese goblin called a *tengu* 天狗 (a divine being in Japanese folklore). During the Edo period (1603–1867), Akiha Sanjakubō was widely revered as having control over fire. Akihabara 秋葉原, the neighborhood of Tokyo where the collective bought electric parts for MaCA’s installation, is renowned globally for anime and subculture, and derives its name from the Akiha Gongen. Legend has it that the technique for extracting thread from kudzu was developed by a Shugendō practitioner—a mountain ascetic who combines esoteric Buddhist, Shintō, and Taoist practices through rigorous physical and spiritual training in nature— in Kakegawa, Shizuoka prefecture, not far from another Akiha mountain. ( Fukatsu, 2008, 41) On Takaba Mountain, power lines carrying electricity produced by nuclear power from Niigata to Tokyo stand in areas covered by kudzu. Kudzu is often seen here as both a plant that covers the mountain and a “weed” that invades the power station. Meanwhile, on Gongen Mountain, there is a small stone shrine on the summit dedicated to horses. It is said that long ago, horses frequently rolled down the slopes and died while eating the grass that grew on the mountain, and the shrine was built to mourn their spirits. In this way, the relationship between the souls of the dead horses and humans is inscribed into the landscape. These layered memories of the mountains and plants informed MaCA’s bodily gestures and choreographic translations.

**Figure 8.**
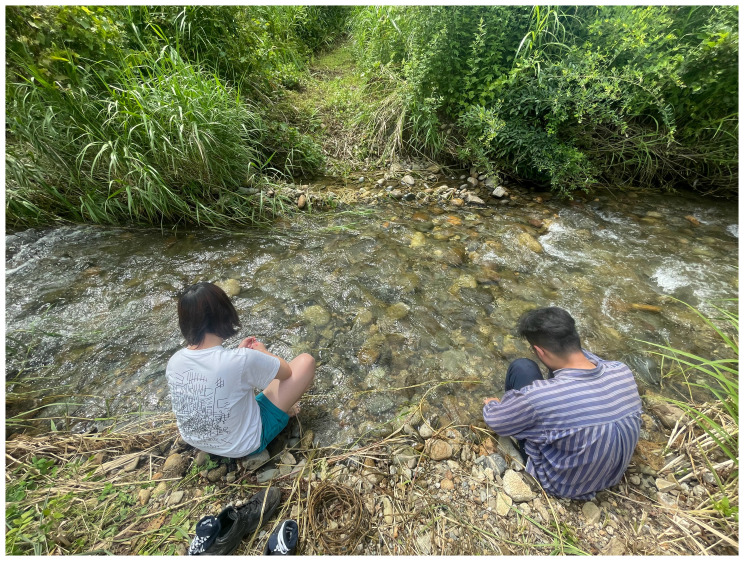
Washing kudzu (Pictured: J. Yamaguchi and M. Maeno). Photo by Shuntaro Yoshida.

**Figure 9.**
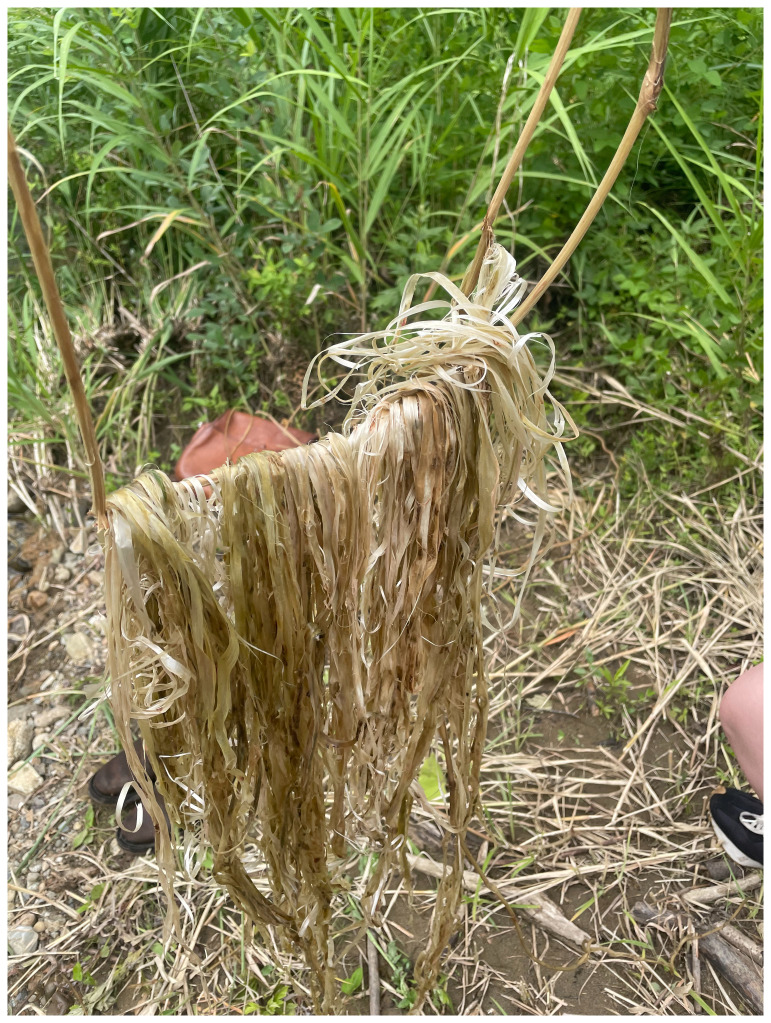
Kudzu fiber. Photo by Shuntaro Yoshida.

Through kudzu movements and the collective’s gestures (boiling, fermenting, and river-washing) which render kudzu into fiber, the collective enacted the traditional kudzu-weaving techniques documented on the Kakegawa crafts website, which also reflects on the loss of craft skills in an era of modernization ( Figure 8 and Figure 9). When translating the movement of kudzu into task-based dance scores—simple written and drawn instructions that guide bodily actions inspired by the plant’s growth patterns—the collective treated kudzu itself as a witness, not in the human sense of an observer, but as an other-than-human presence that records and remembers. In this way, the witness of kudzu links the memory of local territories with the living presence of *satoyama*, the landscape that connects village and mountain. *Satoyama* attunes MaCA to the rhythm of kudzu in Echigo’s ecosystem and debates the symbiosis between humans and non-humans. However, MaCA does not only demonstrate the well-worn observation that “human beings are part of nature,” but also that kudzu and Akiba mountain in their other-than-human personhood echo with the past and future of the life cycle and resist human control—we watch the clumsy-seeming mountains.

## Navigation of the living archives and ecological considerations: *Clumsy-Seeming Mountains*, the installation (Tōkamachi Snow Center)

**Figure 10.**
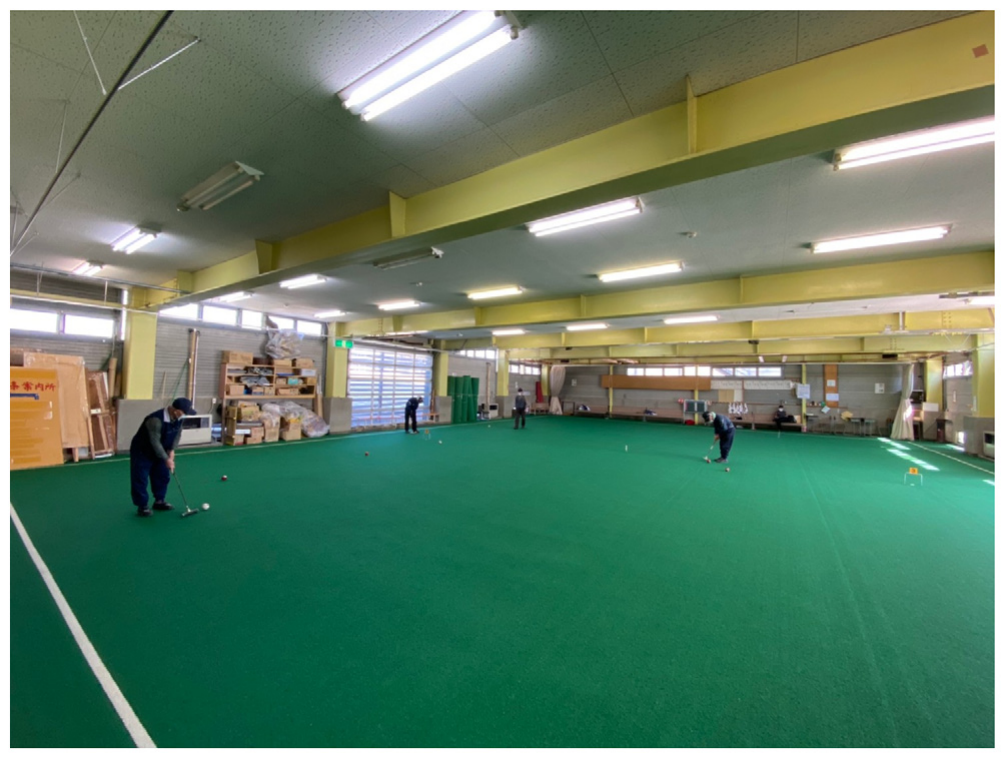
Tōkamachi Snow Center in Winter 2020. Photo by Maharu Maeno.

**Figure 11.**
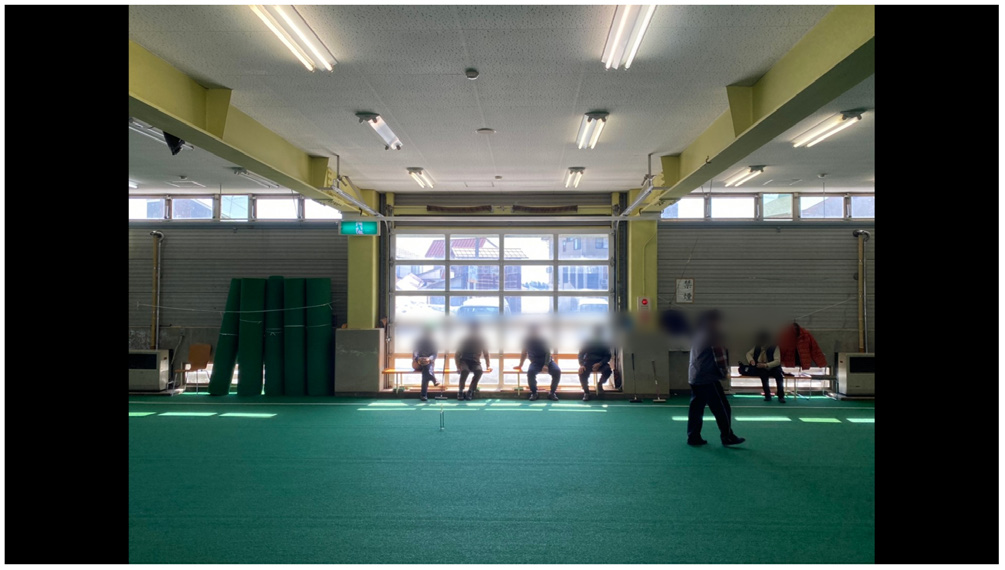
Tōkamachi Snow Center in Winter 2020. Photo by Maharu Maeno. Figure 11 has been reproduced with permission from the participants A, B, C, D, E F and G. Edits were made for de-identification purposes with permission from Maharu Maeno.

In the *Clumsy-Seeming Mountains* installation, MaCA’s installation was housed in the Risetsu Shinsetsu Sōgō Center, Tōkamachi Snow Center (MaCA’s translation), a former bathhouse surrounded by rice fields. In the winter, the two-storey building serves as an emergency shelter and an indoor croquet lawn ( Figure 10 and Figure 11). MaCA’s multimedia archive stood on the field’s artificial grass.

**Figure 12.**
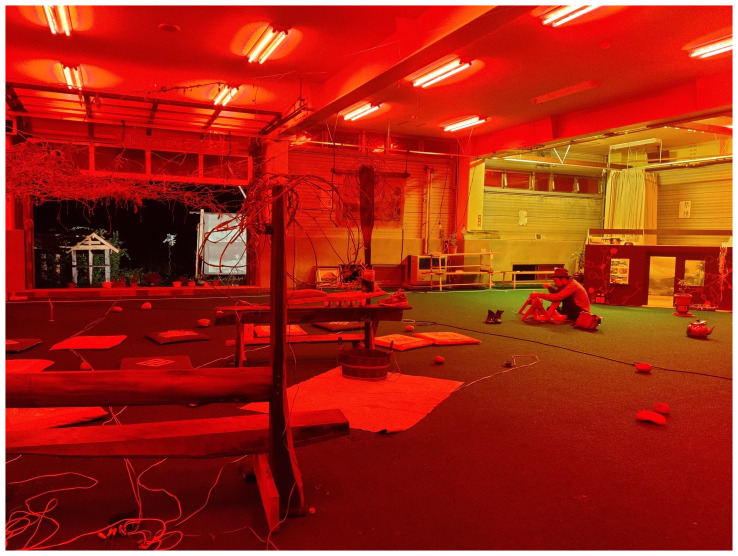
Threading kudzu vines (Pictured: J. Yamaguchi). Behind him is the scaled-down version of *El Arenero Yumita*. Photo by Catalina Fernandez.

**Figure 13.**
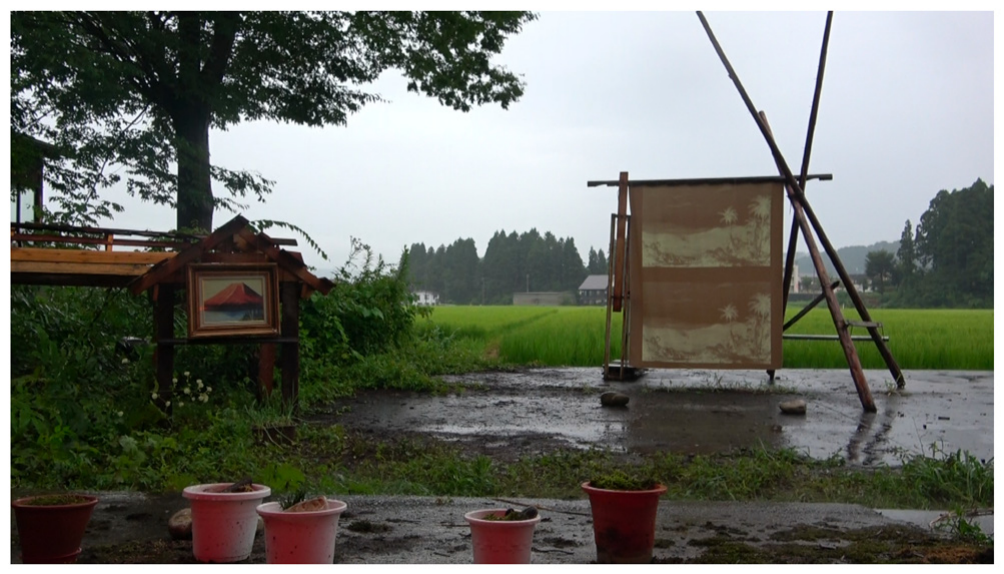
Outside, the scaled-down version of Yamaneko Guest House with a portrait of Mount Fuji and a double reproduction of Frederic Church’s portrait of Mount Cotopaxi. Photo by Julie Lee. Figure 13 has been reproduced with permission from Julie Lee.

**Figure 14.**
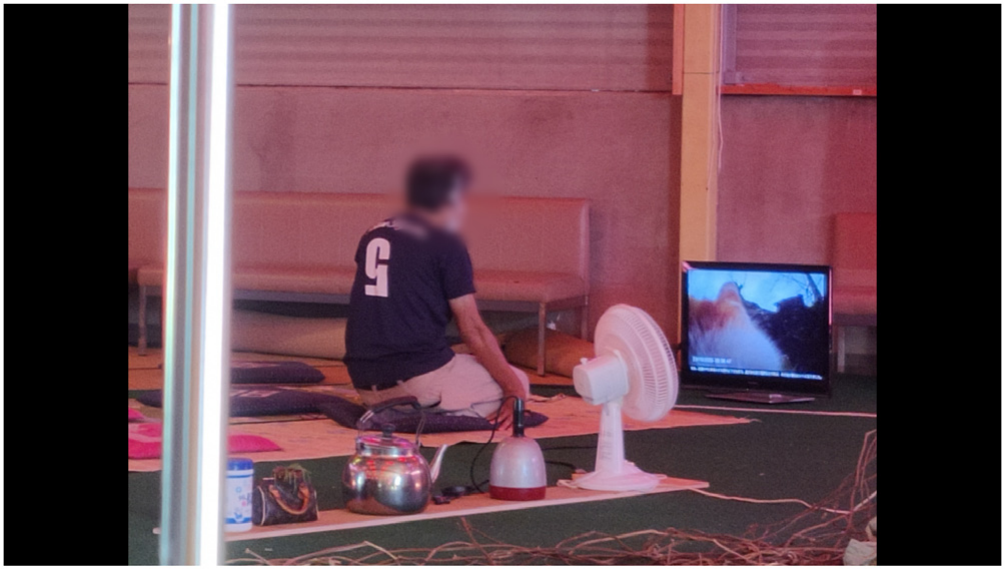
Detail of MaCA’s installation *Clumsy-Seeming Mountains* in 2022. Photo by Catalina Fernandez. Figure 14 has been reproduced with permission from participant H. Edits were made for de-identification purposes with permission from Catalina Fernandez.

The installation emulated a sci-fi bathhouse. MaCA installed red filters in the overhead lamps and windows and played a sci-fi soundtrack composed by Fernandez using sounds from natural environments and electronic noise ( Figure 12). The whole experience bathed the croquet lawn in an eerie red glow, making the familiar landscape feel strangely alien to locals and visitors. The deep red of the interior gave the greens a particular intensity, a visual illusion similar to the water stage in Min Tanaka’s Butō performance, highlighting the presence of rice fields around MaCA’s installation. ^10^ In the field, MaCA placed TVs with its video diaries, documentation of the vine’s entanglements, three-dimensional kudzu-threaded mountains, the aforementioned dance scores, poems, and a model of El Arenero Yumita’s house. Outside, MaCA installed a scaled-down version of Yamaneko’s Guest House and a double print of Frederic Church’s portrait of Mount Cotopaxi ( Figure 13). ^11^ Visitors were invited to explore the grounds barefoot, encounter the trembling mountains, and have a glimpse of MaCA’s video and photographic archives documenting the seasonal growth of its digital-residency garden and the gardening processes in Japan and Germany ( Figure 14).

By installing the aforementioned MaCA’s archives in the Risetsu Shinsetsu Sōgō Center, MaCA aimed to link the collective’s past to the rice fields and the mountains, a living archive just outside of the exhibition space. However, the rice fields slowly change presence outside and humans harvest rice according to the seasonal cycle. Rice fields represent the time-space between nature as wild and as tame. The performance of rice fields enmeshes people in plant agencies. Even though rice fields have been heavily domesticated, they continue to exhibit behaviors that are not solely driven by human desires; rather, they also put on their own performances. Indeed, visitors are aware of the performance of rice fields and watch three huge mountain objects, each about three to five meters in diameter, made by collecting and weaving local kudzu, hung in the space. Mountains made of kudzu in the building and the real mountains outside guided the visitors to in-between perspectives. Mountains were vessels of organisms and living archives in order that visitors walked around inside/outside of mountains, staying close to Earth beings. Thus, the choreography of the clumsy-seeming mountains reopens the discussion of ecological considerations and brings a sense of kinship.

**Figure 15.**
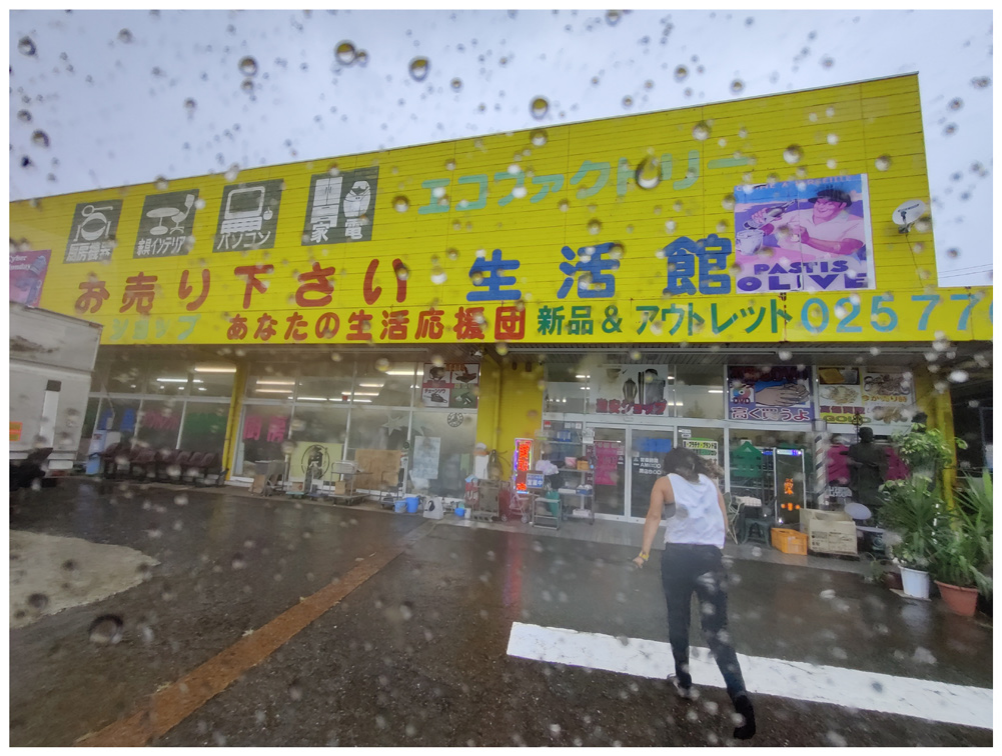
Collecting materials at Eco Factory in Muikamachi, 2022 (Pictured: A. Viteri Arturo). Photo by Catalina Fernandez.

As mentioned above, MaCA managed to use 90% found natural objects and reused materials from the site for both the exhibition and the performance. On the other hand, the sound and lighting equipment involved manufactured plastic products. In fact, MaCA struggled to find second-hand, recycled materials of sound and equipment for the installation. The vast amount of e-waste produced in Japan is a serious concern for the Japanese environmental movement, ^12^ and the use of second-hand materials does not seem to be a common practice. MaCA searched for used headphones, cables, and transmitters in the basement of a second-hand, recycled materials store in Akihabara, Tokyo. The omnipresent of plastic packaging in daily existence in Japan presented a challenge to a collective seeking to adhere to eco-friendly practices ( Figure 15). When dismantling the installation, the newly purchased items were sent to Germany for reuse. Moreover, Maeno was able to avoid discarding the debris and instead preserved it by turning it into ash a living archive.

## An emergent form of ecological performance: *Turn Off the House Lights*, an immersive performance

**Figure 16.**
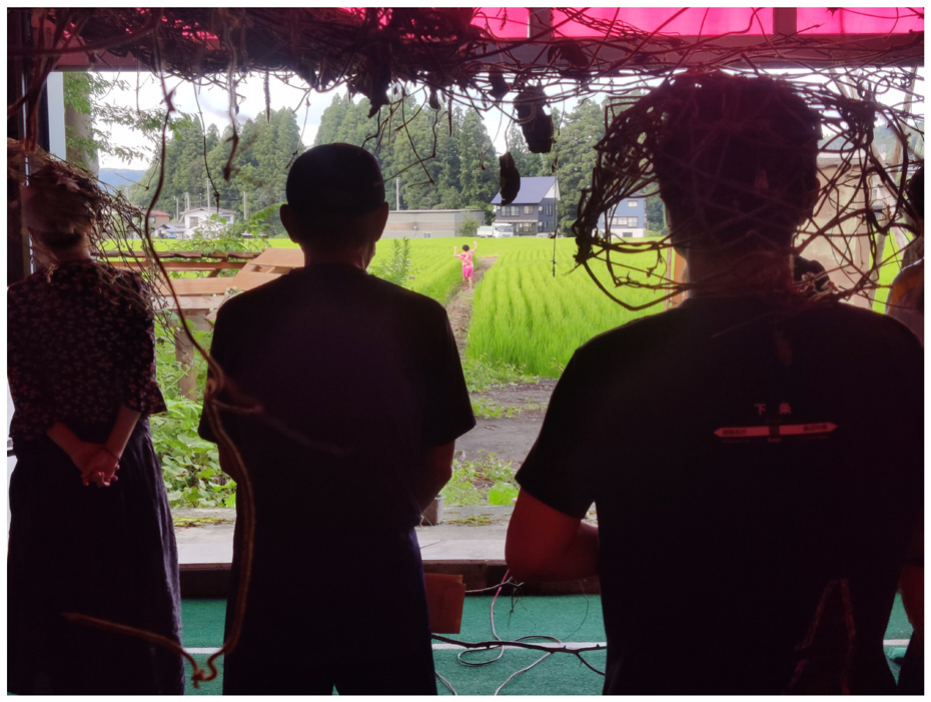
Rice field dance, *Turn Off the House Lights*, 2022 (Pictured: S. Yoshida). Photo by Catalina Fernandez.

During MaCA’s time at ETAT 2022, the collective listened to rice farmers, local volunteers, and sightseers. For its contribution to the exhibition, the collective shared the atmospheric performance *Turn Off the House Lights*, adapting the script to the region’s stories and integrating movements and gestures inspired by the collective’s intra-actions with the landscape and the people. *Turn Off the House Lights* transformed MaCA’s embodied research into a tangible experience, immersing MaCA’s guests in the sensuous realms of real, imagined, and imaginations-of-real landscapes ( Figure 16) ( Tamler, 2022).

**Figure 17.**
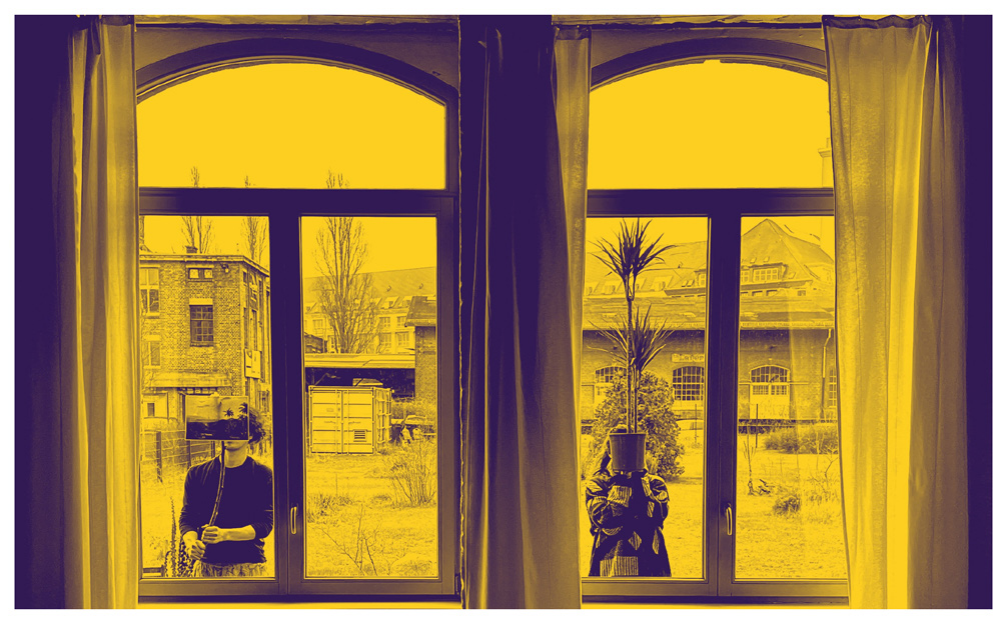
Instagram post, *Turn Off the House Lights* at Cordillera Berlin, 2022 (Pictured: S. Yoshida and A. Viteri Arturo). Photo by Maharu Maeno.

In the Berlin rendition of *Turn Off the House Lights*, the collective guided guests wearing wireless headphones from a dance studio to a horticultural garden, stewarding them through an imagined forest ( Figure 17). The outside community garden features several islands of flowers and plants cared for by neighbors. Together, the collective meandered—something like taking a walk while chatting on the phone with a loved one or sitting with a group of friends to listen to a thunderstorm—before convening around a bonfire to witness the sunset. MaCA’s narrations layered Tokyo’s urban views with Frederic Church’s portrayal of the Andean mountain chain. ^13^ MaCA’s script aimed to challenge colonial interpretations of the landscape and delved into the mountains’ spiritual lives ( de la Cadena, 2015, 25).

**Figure 18.**
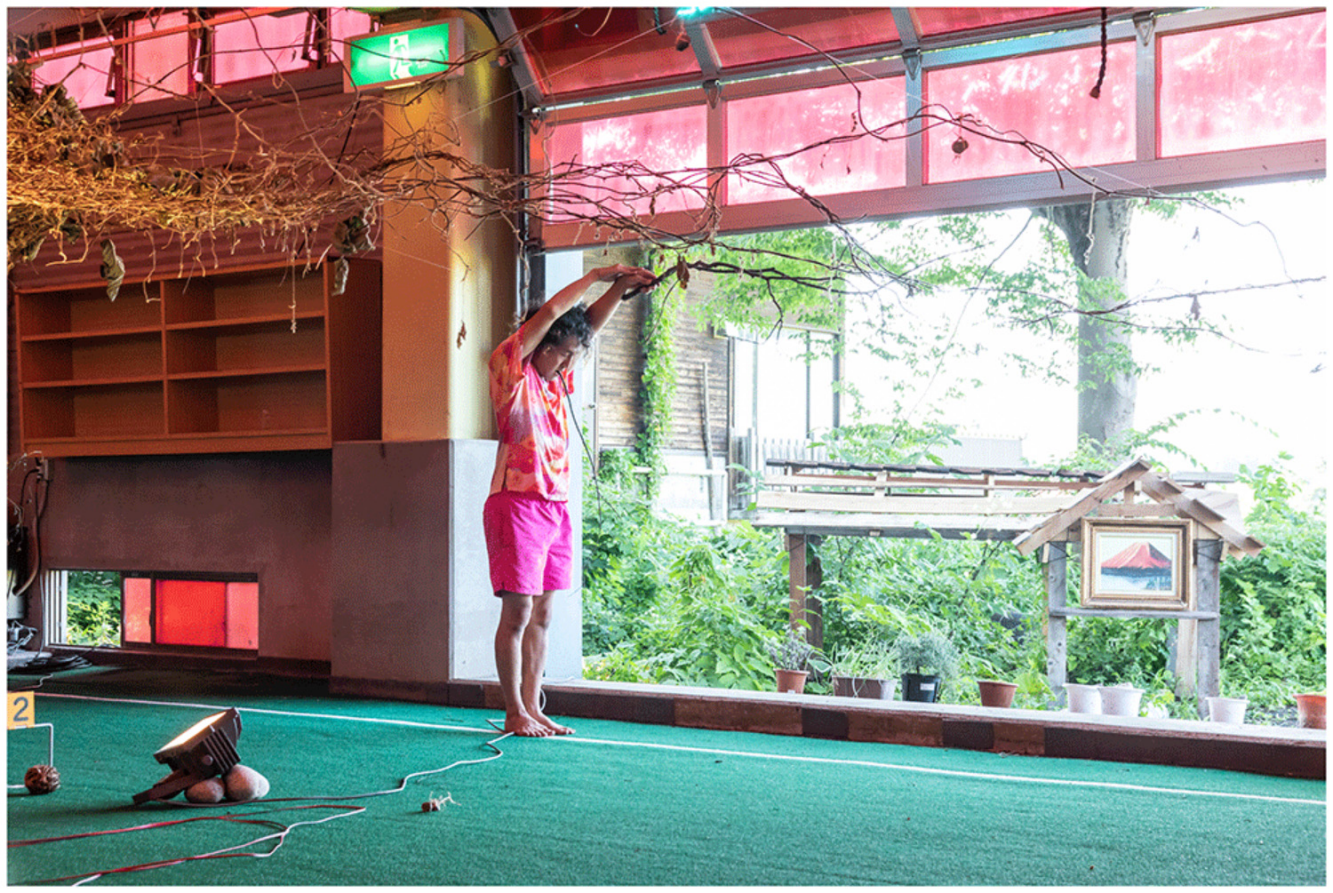
Cherry tree beside the river in *Turn Off the House Lights*, 2022 (Pictured: S. Yoshida). Photo by Osamu Nakamura. Figure 18 has been reproduced with permission from Osamu Nakamura.

Conversely, the Japanese version incorporated embodied renditions of the collective’s visits to Takaba mountain, gestures developed while handling kudzu, and stories from local rice field farmers. MaCA wanted visitors to have an immersive experience that reflected the experience of viewing Mount Fuji while submerged in a hot spring, part of Japan’s bathhouse culture. While Maeno and Arturo told stories, Fernandez mixed MaCA’s soundscape live, Yamaguchi sewed kudzu fibers, and Yoshida performed non-human choreographies – he became a spider in a bathhouse, a bird in a rice field, the pine tree in Church’s paintings, a cherry tree beside the river in Kōtō-ku, a kudzu vine, and one stalk among the many in the rice fields. Emerging from the back of the rice fields and returning to them towards the end, Yoshida describes the experience as the following of disobedient movements, something in between the wildness of the mountains and the domesticated nature of the fields ( Figure 18):

- I tried to draw every movement from the environment. I moved in response to the wind, the clouds, the weaving of the rice fields, followed mountain ridges, and channeled distant rainfalls. The more I was invested in their movement, the lighter my movements became. In the in-betweenness of domestic and wild, the body’s senses did not know which nature it was responding to. ^14^

In the performance of *Turn Off the House Lights*, the rhythms of the body and nature resonate through the sensory and perceptual (re)organization induced by other-than-human persons’ choreography. To “perform the other-than-human” here means to assemble and improvise with multiple other-than-humans’ scores in real time—each score deriving from observed growth patterns, material resistances, or site conditions. Human and non-human bodies do not coincide; misalignment inevitably produces movement in malfunction. Crucially, this gap is not an error but a register of non-coincidence: when a performer aims at the land and yet clearly fails to “match” an other-than-human choreography, a trace of empathy remains without erasing other “aliens”. Dance scholar Tamara Ashley’s proposal for ecological choreography introduces and expands upon the notion of performances that are primarily linked to place ( Ashley, 2012, 27). Going against this, dance and theatre writer Maša Radi Buh explains that the location is sequentially changed and updated in trans-geolocation choreography through the sound walk. She mentions the updated ecological choreography in the following:

- Ecological choreography not only incites an ecological experience of awareness through theatrical content but offers an experience through the medium itself as well. The difference between the former and the latter is that one of them counts on the audience’s feelings while the other engages them with embodied ways of attending and perceiving. ( Buh, 2022, 47)

Buh proposes a geolocational multimedia soundwalk as ecological choreography. Such a performance engages the attendee through a physical attunement that is connected to two interpretations of listening, both connected to artistic practices aiming to aid the body in experiencing some type of ecological awareness. Participants listen to a set of audio tracks which correspond to certain locations along the route, with intra-connected elements giving the sense of a unified event. She describes the connection between the three elements of space, time and sound, observing that this connection “exemplifies the principle of intra-connectedness taken over from ecology as the change or fault in the execution of one of them significantly alters the performance itself, as well as the participant’s experience” ( Buh, 2022, 47–48). Buh’s ecological experience of awareness indicates attentive walking and recalls the absence of the noises of civilization during the COVID-19 pandemic. In MaCA’s performance in Berlin, the collective challenged the soundwalk, inviting gardens into an embodied practice. However, simply told, MaCA’s performance at ETAT 2022 couldn’t avoid such theatrical frameworks such as the division of space between stage and auditorium space because MaCA couldn’t use remote headphones in Japan. ^15^ During the performance in ETAT, guests were located between live narrators (behind guests) and performer, and the fixed location prevented the changes and contingencies of participants moving the environmental structure.

## Conclusion

In this case study, MaCA has presented its collective research-creation and *Turn Off the House Lights*, a work of other-than-human choreography. The choreography navigated the border zone between human and non-human, which emphasized ecology territories inciting an ecological experience of awareness. Rather than centering subjective experience, MaCA’s account traces the processes and relations through which the work unfolded: attuning to site-specific ecologies, following entanglements among beings and materials, and translating these encounters into task-based scores and installations. MaCA understand movement in malfunction as a resonant movement that emerges from the intra-connected of human motions—arising in contact with environments and other-than-human persons—and movements generated by other-than-human choreographies. Within MaCA’s living archival practice, other-than-human choreography operates intra-disciplinarily across visual art, music, lighting, dance, theatre, and text. The back-and-forth of incommensurable noise—movement in malfunction and disobedience movement inherent to Earth’s beings—does not interrupt the work; it conditions it, offering methods for sensing kinship, recalibrating relations, and responding to future ecosystems.

**Figure 19.**
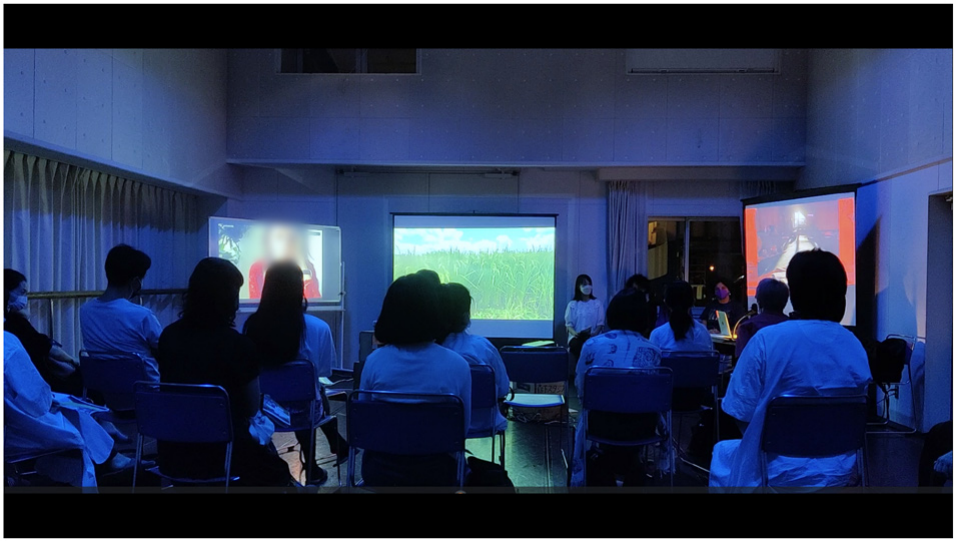
“広い島/HIROSHIMA NO TIENE NADA QUE ENVIDIARLE A PARIS,” hosted by The Saison Foundation, Tokyo, 2022 (Pictured M. Maeno and C. Fernandez). Photo by Taro Inamura. Figure 19 has been reproduced with permission from Taro Inamura. Edits have been made for de-identification purposes with permission from Taro Inamura.

Writer, translator, and interdisciplinary artist Cory Tamler interviewed MaCA at Morishita Studio after the performance of *Turn Off the House Lights* at the ETAT ( Figure 19). She wrote of MaCA’s work that:

- the research and creation process is one of shared archiving, a kind of living accumulation – analogous, in my mind, to the simultaneous growth and decomposition in a garden. The collective (de)composes their archive through the exchange of gardening techniques and a “practice of encounter” (in their words) with non-humans, but also through their varied artistic practices, from sound and visual art to dance. ( Tamler, 2022)

The collective’s living archival practices are comprised of the transmission and performance of other-than-human persons’ lives and forms.

From digital residency and site research to installation and performance, the collective’s living archives accumulate, on the one hand, the conceptual malfunction that spills over from material exploration. The malfunctions that appear during the creative process address phenomena beyond the human and open the way for an escape from the anthropocentric perspective. On the other hand, a feeling of substantive disobedience spills over from transcendental exploration. The disobedient movement of other species dissolves the dichotomy of human and non-human, and changes to the tuning of their rhythm in an intentional break from the dramaturgical framework. The collective’s senses shifted back and forth in the encounters with other-than-human persons’ choreography, underlining the way other-than-human approaches reflect the complex relationship between humankind and the natural world. However, living archival practice in a collective life can contribute to concern and care for both the Earth and the disobedient movement of its living beings. This practice helps sustain the collective lives of other-than-human persons and their ecosystems.

## Suggestions for the future research

At the time of writing, MaCA’s artistic practice only incites the consciousness of environmental awareness. Furthermore, considering the Earth’s rapid deterioration over a decade since 2000s, it is significant that MaCA considers how artistic works represent ecological choreography. From the perspective of the carbon footprint, MaCA might also have to reconsider the use of air travel in order to sustain an ethical engagement with the ecosystems to which its choreography responds. Despite the development of theories of the Anthropocene and posthumanism, these have primarily been applied to science and artwork rather than intra-disciplinary research. Thus, MaCA argues for a more radical development of embodied practices and interactions between human and non-human environments to elicit a new investigation of the relationship between the human and the non-human that may be experienced by audiences. Most importantly, intra-disciplinary perspectives should transform collaboration in the collective through encounters with other-than-human choreographies, opening the way to further living archival practices.

Finally, it is essential to note that the Institutional Review Board (IRB) confirmed that no formal ethical review was required for this research. Nevertheless, because MaCA deliberately refers to its collaborators as other-than-human persons, the project implicitly challenges anthropocentric notions of ethics and consent. Even when no additional approval was deemed necessary, such terminology compels researchers to consider what ethical stance is being taken toward non-human collaborators—the potential risks, forms of care, and accountability involved in recognizing them as agents or persons in the research and creative processes.

## Ethics and consent

Ethical approval and consent were not required. The authors confirm that consent to publish the name of Luz Marina Giraldo. Any identifiable information has been used with explicit permission for publication. Regarding the consent to publish identifiable details, written informed consent was obtained via her family member because she has passed away.

## Data Availability

No data is associated with this article.
