# Supplementary material for: Living archival practice and the choreographical navigations: Encounters and approaches with other-than-human persons
Source: Open Res Eur. 2025 Nov 29;4:171. Originally published 2024 Aug 7. [Version 2] doi: 10.12688/openreseurope.17447.2 (PMC11579586; doi:10.12688/openreseurope.17447.2)
Supplement: Peer review history

# Reviewer report: Reviewer response for version 2

**DOI:** 10.21956/openreseurope.23774.r65099

**Published:** 2025-12-23

**Authors:** Ian Garrett

**Affiliations:** 1 York University, Toronto, Ontario, Canada

The restructuring and placing more contextual information earlier in the paper are great improvements to understanding the relationship of the pieces to one another and the timeline for the work.

Is the case presented with sufficient detail to be useful for teaching or other practitioners?

Yes

Is the work clearly and accurately presented and does it cite the current literature?

Yes

If applicable, is the statistical analysis and its interpretation appropriate?

Not applicable

Are all the source data underlying the results available to ensure full access and reuse by other researchers?

Partly

Are the conclusions drawn adequately supported by the data and analysis?

Partly

Is the background of the case’s history and progression described in sufficient detail?

Partly

Reviewer Expertise:

Extensive research/creation experience focused on ecological design for performance or "ecoscenographic" approaches to performance and time-based art publishing across Theatre, Dance, and Performance Studies and Digital Environmental Humanities with a focus on extended reality technologies in site-specific and geolocated performance projects and the ethics of this work, in part through non-human collaboration working both as an international touring artist, and through a variety of peer-reviewed publications. Educator focused on ecoscenography, devised and site-specific performance and research creation supervising graduate students on related project as former director of graduate program of Theatre, Dance, and Performance studies.

I confirm that I have read this submission and believe that I have an appropriate level of expertise to confirm that it is of an acceptable scientific standard.

# Reviewer report: Reviewer response for version 2

**DOI:** 10.21956/openreseurope.23774.r65100

**Published:** 2025-12-11

**Authors:** Teresa Heiland

**Affiliations:** 1 University Of North Carolina, Greensboro, North Carolina, USA

Dear editors,

 The paper continues to improve, but it remains rather dense and difficult to follow at times. I have some new recommendations that I wish I had sent along in the first round. Please forgive the delay, but I think these revisions will help the paper to allow a reader to sustain focus. Here are some recommendations to support more focused engagement among readers. These changes are not complex, but will provide anchors for readers:

**1. The article is very dense in its opening section.** To increase readability, consider breaking the introduction into smaller subsections (e.g., “Anthropocene Debates,” “More-than-Human Performance,” “MaCA’s Positioning”) to help readers track the conceptual stakes.

**2. I think a Method section is needed:** The ethnographic detail of MaCA’s process—particularly the digital residency, kudzu research, and ETAT site practices—provides a textured sense of the collective’s methodology. However, t **he article would benefit from a clearer articulation of methods** **.** A concise methodological section early in the paper would strengthen its academic ground.

 For example:

- **What were the research questions** guiding the digital residency? Can these questions be stated early in a method section to help readers to follow the author’s line of thinking?
- H **ow was video analyzed**? Did authors use qualitative video analysis software tools such as NVivo, Atlas.ti, and MAXQDA, which help to track themes. What system did they use? Published research should be shared in such a way that another team of researchers could replicate the methods.
- **How were kudzu gestures documented or translated into scores?** I believe some sample scores would reveal the thinking process and creative practice within the methodology. Also, are not the scores artifacts or documents that represent artist-researchers' relationships with the ecosystem?

**3. Please define “malfunction” a paragraph earlier in paragraph 6.** It is used in that paragraph without explanation, so meaning is vague, and it is not described until paragraph 7.

 Excellent work. Interesting paper.

Is the case presented with sufficient detail to be useful for teaching or other practitioners?

Partly

Is the work clearly and accurately presented and does it cite the current literature?

Partly

If applicable, is the statistical analysis and its interpretation appropriate?

Not applicable

Are all the source data underlying the results available to ensure full access and reuse by other researchers?

Partly

Are the conclusions drawn adequately supported by the data and analysis?

Yes

Is the background of the case’s history and progression described in sufficient detail?

Partly

Reviewer Expertise:

Ph.D. in Dance in Higher Education and AdministrationExtensive experience publishing qualitative and statistical research about the artsEditor and creator of an online academic, peer-reviewed journal about dance. Author of a peer-reviewed book and multiple articles on dance topics. Educator of site-specific processes in the arts. Experience with dance in South East Asia and Japan.

I confirm that I have read this submission and believe that I have an appropriate level of expertise to confirm that it is of an acceptable scientific standard, however I have significant reservations, as outlined above.

# Reviewer report: Reviewer response for version 1

**DOI:** 10.21956/openreseurope.18854.r45664

**Published:** 2024-11-20

**Authors:** Natalie Schiller

**Affiliations:** 1 The University of Auckland, Auckland, Auckland, New Zealand

Reading this article might urge the reader to get down on their hands and knees to re-experience the world from an *other-than-human* perspective (at least this is what happened to me!). In this case study, the authors celebrate the propositions of *other-than-human* choreographies and ecological choreographies. The authors are part of the international and interspecies collective *Mapped to the Closest Address (MaCA)* and cartograph four events of their research-creation in this writing. Here, they introduce their insights from their digital residency ( *Open Forest Launch*), site-research, installation ( *Clumsy-Seeming Mountains)*, and immersive performance ( *Turn Off the House Lights*). Their work intra-mingles *earth beings*, such as a cat, kudzu vines, and mountains ( *satoyama*), and *other-than-human* performative actions, such as processes of fermenting. O *ther-than-human *entanglements stimulate ideas on *disobedient* and *malfunctioning* movements. The choreographies seem to emerge in “zone[s] of indiscernibility” (Massumi, 2014, p. 8), where notions of the “domestic” and the “wild” (article p. 9) are con-fused.

 This case study is well written and allows the reader to encounter a *tacit *and *felt* knowledge that research-creation seeks to offer (Loveless, 2019; Nelson, 2022). This writing is a unique and significant contribution to the realm of research-creation and captures essences of the concepts on how and when assemblages of *other-than-human* beings and ecological choreographies can activate artistic practices.

 With a focus on *other-than-human beings*, I encourage the authors to re-evaluate the chosen words and if those terminologies mirror the dynamics of their research-creation they seek to express with this case study. For example, the term “other-than-human persons” has mainly been applied throughout the case study, although on page 10 the word person has been left out, creating “other-than-human choreography” *, *which for me captures the particularities and core of this research-creation more specifically.

 Furthermore, could the integration of *intra* (Barad, 2007) emphasise notably the ethos and dynamics of the *more-than-human* configurations within the case study? Barad’s agential realism thrives on its neologism of *intra*, where *intra* ” *signifies the mutual constitution of entangled agencies*….[and] *agencies are only distinct in relation to their mutual entanglement; they don’t exist as individual elements*” (p. 33). Therefore, the authors could consider implementing instead of *inter* or *cross* an *intra*. For example, interconnectedness (p. 2, 3, & 10) could morph into intra-connectedness, interactions (p. 2, 3, 9, & 11) could transform into intra-actions, and cross-disciplinary (p. 11) could be intra-disciplinary.

 On another note, the work of Katja Aglert et al. (2024) might be of interest to the authors.

Is the case presented with sufficient detail to be useful for teaching or other practitioners?

Yes

Is the work clearly and accurately presented and does it cite the current literature?

Yes

If applicable, is the statistical analysis and its interpretation appropriate?

Not applicable

Are all the source data underlying the results available to ensure full access and reuse by other researchers?

No source data required

Are the conclusions drawn adequately supported by the data and analysis?

Yes

Is the background of the case’s history and progression described in sufficient detail?

Yes

Reviewer Expertise:

My areas of expertise lie within artistic research, feminism, performance arts, performance philosophy, and posthuman/new materialism theories. My projects seek to explore how artistic research supports critical, creative, and affirmative approaches of becoming and being in a contemporary world. Some of my work has been published via the following articles: (2020) ‘Experiments in Motion – An entanglement of dancing hips and domesticity.’ RIACT – Journal of Artistic Research, Creation and Technology, 1(1), 51-75, (https://researchspace.auckland.ac.nz/handle/2292/54430) and (2017) ‘Breathe through your vagina! — An attempt to catch ineffability.’ Dance Research Aotearoa, 5(1), 47-58 (https://doi.org/10.15663/dra.v5i1.60). I am a member of the international posthuman reading and writing group, Posthumanism in/as Artistic Practices, and associate editor with The International Journal of Education and the Arts.

I confirm that I have read this submission and believe that I have an appropriate level of expertise to confirm that it is of an acceptable scientific standard.

## References

[1] : Feminist Reconfigurings of Alien Encounters.2024; 10.4324/9781003373766 10.4324/9781003373766

[2] : Meeting the Universe Halfway.2006; 10.1215/9780822388128 10.1215/9780822388128

[3] : What Animals Teach Us about Politics.2014; 10.1215/9780822376057 10.1215/9780822376057

[4] : Practice as Research in the Arts (and Beyond).2022; 10.1007/978-3-030-90542-2 10.1007/978-3-030-90542-2

[5] Reference source

# Reviewer report: Reviewer response for version 1

**DOI:** 10.21956/openreseurope.18854.r45661

**Published:** 2024-11-19

**Authors:** Stefanie Gabriele Sachsenmaier

**Affiliations:** 1 Middlesex University London, London, England, UK

The article documents the artistic practice of the dance collective Mapped to the Closest Address, outlining a series of its projects since 2020. It discusses the collective's creative processes and methods, thematising issues such as human and other-than-human relations, disobedient movements, interconnectedness between human and more/other-than-humans, entanglement, ecological awareness etc. 

 The strength of the article is its perspective of the makers who provide insights into the creative processes and critical approaches explored. It includes a strong resource of visual materials, which convey the artistic practices in a mixed mode to the reader. 

 What might be further considered in developing the work is its rather long list of keywords, partially listed above. While these are very current terms and there is a sense of how these belong together, as such these concepts remain largely undefined and under-explored. Indicatively, questions open up along the lines of what constitute practices of disobedience and in what ways the documented practice might align and perhaps also expand these. 

 Furthermore, the article comprises a long list of footnotes, which, if to be read in line with the main text, create a disruption to the flow of reading. While some of these materials do indeed work well in footnote form, others provide details that seem key to understanding the thinking that is developed in the main text. It would be helpful if the authors considered to place some of the materials presented in footnotes into the main body of the writing, where the respective ideas further outlined seem vital to the understanding of the discussion.

 I would also point to an opportunity for further criticality of the collective's practice in view of considering its practice in view of ecological practices, such as for instance critically commenting on the carbon footprint it incurs. A slightly more self-critical view would also help to frame the writing as a mode of ongoing enquiry  into the rich, and crucially challenging, ideas and concepts presented, rather than presenting these as having been achieved in their artistic practices.

Is the case presented with sufficient detail to be useful for teaching or other practitioners?

Partly

Is the work clearly and accurately presented and does it cite the current literature?

Yes

If applicable, is the statistical analysis and its interpretation appropriate?

Not applicable

Are all the source data underlying the results available to ensure full access and reuse by other researchers?

Yes

Are the conclusions drawn adequately supported by the data and analysis?

Yes

Is the background of the case’s history and progression described in sufficient detail?

Yes

Reviewer Expertise:

Performance practice, performance processes, process philosophy, posthumanism, performance and politics

I confirm that I have read this submission and believe that I have an appropriate level of expertise to confirm that it is of an acceptable scientific standard, however I have significant reservations, as outlined above.

# Reviewer report: Reviewer response for version 1

**DOI:** 10.21956/openreseurope.18854.r45666

**Published:** 2024-11-12

**Authors:** Ian Garrett

**Affiliations:** 1 York University, Toronto, Ontario, Canada

The article by Yoshida et al. explores the interspecies dance collective, Mapped to the Closest Address (MaCA), and their approach to choreography with other-than-human persons. It details their living archival practices from a digital residency during the COVID-19 pandemic to performance installations at the Echigo-Tsumari Art Triennale 2022. The collective incorporates non-human elements like a feline collaborator, kudzu vines and mountains into their work, aiming to break down human-centric views and foster an ecological awareness through emergent and disobedient choreography.

 The article presents an innovative and compelling approach to ecological and environmental site-specific choreography, emphasizing interactions between humans and non-human entities. It successfully introduces concepts like “malfunction” and “disobedient movement,” which challenge traditional human-centered choreography practices. The analysis of MaCA’s performances and installations provides a rich narrative, drawing attention to the interconnectedness of human and non-human-collaborators.

 There are areas where the clarity and organization of the paper could be improved:

 Terms such as “malfunction,” “disobedient movement,” and “other-than-human persons” need clearer initial definitions, at least to understand the use by the authors. While they’re well considered and appropriate, the unfamiliar reader would benefit for help in understanding how these are being used more explicitly. Introducing these concepts earlier with brief definitions on what is meant by their use in the paper would help readers understand the framework without having to refer to later sections or accept ambiguity with their own understanding.

 I think it would be useful to also unpack the focus on the idea of the “non-human person”. While collaboration with non-human entities or non-human collaborators has become relatively common in at least some conversations, there use of “person” is an interesting divergence and may load the reader’s understanding with a specific idea of the relationship between humans and non-humans as people, which feels like it can pull the efforts of the authors to explore the non-anthropocentric views of their non-humans by suggesting their personhood and the social relationships and rights of personhood in anthropocentric frameworks. For this reviewer, it pulled the understanding of the relationship to each of the non-human collaborators towards the anthropocentric and may undermine the idea. Further to this, it also feels like the authors would benefit from the addition of writing on their thinking about the ability of humans and non-humans to share experiences given the differences is physiology, neurology, and each’s distinct evolved sensorium.

 There are allusions to this consideration, such as the citation of Frickhorn on pg. 3. I think this is particularly important in the description of Shutaro’s expression of non-human choreographies on pg. 9, where I’m curious to hear about the introduction of human reasoning and creative decision making in translating the non-human to the human and what sort of fidelity is possible and what further mutual understanding is found in recognizing these uncrossable boundaries between human and non-human experiences of the world.

 As an important distinction stemming from this thinking, when it comes to the Ethics and Consent section at the end, It would be helpful to confirm that the fact that Ethics approval was not required was confirmed by an IRB. Given the choice to refer to collaborators as “non-human persons” there is a question of the anthropocentric concept of ethics and consent in a research context. If the non-human collaborators are being given personhood, then it stands to reason that there would need to be consider of the risks and potential harms to the non-humans and associated ethical approaches. This reinforces the need to be explicit and intentional about the use of this term. So, while an IRB may not require further consent and indicates it does not require further approval based on institutional risk, what are the ethics of the researchers if non-humans are positioned as persons?

 The article would also benefit from restructuring certain paragraphs to improve coherence. For example, the section discussing kudzu vines and mountains could be presented in a way that better integrates the description of site-specific practices with the underlying theoretical concepts.

 It may also be useful to add an early paragraph with a clear timeline of activities along with their locations, and how other locations are layered. For example, on pg. 9, there is reference to the Berlin iteration of the work, which was a location less clear to me as a site of performance early. This discussion the layering of Tokyo over the experience, and I was unclear about how these geospatial relationships were being described. The following paragraph then describes the Japanese version, which presents some confusion given the scale of the location and the previous description of a specific city in Japan being layered over an experience in Berlin. This may also be useful as chart/matrix as an additional figure or appendix to provide the reader with a clear understanding of how the authors layered locations and to offer easy reference to the spatial context of each iteration.

 Overall, the concepts are expressed well. It may be worth addressing dangling modifiers and eliminating colloquial expressions which would make the text more polished. Please also use surnames consistently. There are many specific and excellent suggestions by the previous review which I will suggest the authors heed instead of listing them again.

 It would primarily benefit from providing greater clarity for the unfamiliar reader, so that specific terms and the temporal and spatial planning of the performance work is clear so that the focus is on the author’s critical understanding of their findings, which are somewhere clouded by these ambiguities. Most importantly, a clear understanding, and consistency in the language used to refer to non-human components and either some additional writing on why the authors have positioned non-humans in this way and/or acknowledgements of the limits of human understanding of non-human perspectives would be beneficial to reinforce the de-anthropocentric goals of the paper and the work it described.

 I would suggest that the authors may consider Una Chaudhuri’s *The Stage Lives of Animals* to develop a deeper critical approach to the complexities of non-human expression in performance.

Is the case presented with sufficient detail to be useful for teaching or other practitioners?

Yes

Is the work clearly and accurately presented and does it cite the current literature?

Yes

If applicable, is the statistical analysis and its interpretation appropriate?

Not applicable

Are all the source data underlying the results available to ensure full access and reuse by other researchers?

Partly

Are the conclusions drawn adequately supported by the data and analysis?

Partly

Is the background of the case’s history and progression described in sufficient detail?

Partly

Reviewer Expertise:

Extensive research/creation experience focused on ecological design for performance or "ecoscenographic" approaches to performance and time-based art publishing across Theatre, Dance, and Performance Studies and Digital Environmental Humanities with a focus on extended reality technologies in site-specific and geolocated performance projects and the ethics of this work, in part through non-human collaboration working both as an international touring artist, and through a variety of peer-reviewed publications. Educator focused on ecoscenography, devised and site-specific performance and research creation supervising graduate students on related project as former director of graduate program of Theatre, Dance, and Performance studies.

I confirm that I have read this submission and believe that I have an appropriate level of expertise to confirm that it is of an acceptable scientific standard, however I have significant reservations, as outlined above.

# Reviewer report: Reviewer response for version 1

**DOI:** 10.21956/openreseurope.18854.r44731

**Published:** 2024-10-08

**Authors:** Teresa Heiland

**Affiliations:** 1 University Of North Carolina, Greensboro, North Carolina, USA

**Answers to questions:**

1. Is the background of the case’s history and progression described in sufficient detail?

The study’s background history and progression are mostly clear. I did find one paragraph on page 7 that, if brought far earlier in the paper, would improve reader’s comprehension of the work. I have noted it below. I have also gathered a list of many syntactical and word-choice needs in the writing that could be improved to ensure reader understanding.

1. Is the work clearly and accurately presented and does it cite the current literature?

The work references the theme and progression of the activities. The well-known literature of Anna Halprin and Lawrence Halprin might be of interest to this group. Anna Halprin’s work was about relating to nature. Her husband Lawrence Halprin created gardens for visitors or dances. They both also wrote scores and related to nature in ways that spiritually parallel your work. Lawrence Halprin’s RSVP cycles, page 3 might be a place to start reading. Their work might inspire these authors. Literature seems sufficient, although some of it comes far later in the paper than what is traditionally done. This choice of adding literature later than the way most academic papers read causes the flow to feel less focused and directed than it could.

1. If applicable, is the statistical analysis and its interpretation appropriate? NA
2. Are all the source data underlying the results available to ensure full reproducibility? To have full reproducibility, a list of items used in each of your projects might be helpful. Would a map of the locations be useful as well? For example, how far into natural areas did you travel? People unfamiliar with Japan might not be able to picture the locations without reference points. We will have to Google the locations ourselves. I’m not implying these lists and maps are needed but I think in
3. Are the conclusions drawn adequately supported by the results? Yes.
4. Is the case presented with sufficient detail to be useful for teaching or other practitioners? Some concepts need to be fleshed out more. See my notes below. Also, the word posthumanism is mentioned in footnote 1, but not in the text until the last page (page 11). I would double check to make sure you have fleshed out all the key terms early to introduce the philosophies and concepts early.

**Approved with reservations:**

 The article needs some clarifications in the writing, but it is getting close to being ready. The main idea is strong, but assumptions are made regarding some main ideas that need to be addressed. Just the syntax, grammar, and writing clarifications need to be worked on. Then I think this paper is almost ready.

**Suggestions for improving and polishing the rough draft**:

**Page. 1.**

 In the list of authors’ names after the title, the group title “Mapped the Closest Address” is likely a mistake. The people created the article, not the performing group.

**P.2.**

 Small detail: I see in the abstract that MaCA (which is a group of people but is also a singular entity) is then described as “their.” Consider whether the MaCA should be listed as singular or plural. It seems like a group (singular) when you speak about it as a whole. The grammar was a bit confusing due to word choice.

 The word “this” is a dangling modifier and should be followed by another word. “This ____process/practice/investigation? ____ includes….”

 I see you stated, “The article discusses.” An article is not a living being and cannot discuss anything. I urge you to instead state something like this, “The authors discuss…”

**P.3. Introduction:**

 Question: Is the cat a non-human person? You don’t suggest it as such, but maybe you will later.

 Again, I see you stated, “The article draws.” An article is not a living being and cannot draw anything. I urge you to instead state something like this, “The authors draw…”

 The syntax is not working in this sentence. “Furthermore, the choreography values “a (utopian) idea of a blurring of the distinction between (the value of) human and non-human creatures appears.” (Buh, 2022, 48).  Remove the word “appears.”

 I imagine you will go on to explain what “ *malfunction and disobedient movement” *are; however, I am desiring an explanation here when you first introduce it.

 Footnote 1: Dangling modifier again. See “This is….” What is “this?”

**p.4.**

 I noticed you use the first names of the contributors rather than the surnames: Maharu, Shuntaro, Catalina, and Alex. This choice may be a way to make the article seem personable; however, typically contributors are addressed in articles by their surnames. *Yoshida, Arturo, Fernandez, and Maeno.*

 “Catalina’s mother” should probably be changed to “Fernandez’ mother.”

**p.5.**

 Which “province” did you travel to? I think you might need to state it. You called it “the province.”

 You mentioned, “ *our mountain portraits*” but we don’t know what those are. Can you explain?

 Does the phrase “ *non-human choreographies*” imply that the kudzu is creating itself? What is the role of the human in this choreography?

**p.6.**

 Are the “ *tips*” the root tips or the leaf tips?

 The word very is discouraged in English. The use of the word vigorously alone is more powerful. *very vigorously*

 If you “ *we unravel the composition of kudzu.*” Isn’t this a modernist approach to affecting nature? Maybe you need to describe that the kudzu guided your movement, and it was the choreographer.

 Party of this sentence is sounding vague. Can you clarify? Why is “the acceptance of the mountains” followed by the words, “the Earth beings?” I am not sure what you are implying there. Can you explain a bit more?

 What are “ *multispecies stories?*” You don’t explain this concept and it will be unknown to readers.

 What is a “ *Shugendo practitioner?” *Be sure to describe so readers don’t have to Google it to understand this.

 I think this sentence should start your paragraph, not end it:

*“The mountains transmitted knowledge to us, and we were tuning in to their rhythms rather than the other way around.”*

*What does the phrase, “encountered a composition of kudzu” *mean? Did the composition/design emerge from your efforts, or did you encounter it as you walked through the woods? The word “encounter” is more often about a happenstance meeting with something along a path. I think your use is different, so maybe find a different word.

 What type of dance scores are you referring to in this passage? “When we translate the kudzu movement into dance scores.” Can you clarify the type of dance score? There are so many in existence. Is it written on paper?

 Is “ *the witness of kudzu” *any person who happens to be watching the activity?

 In the section titled, Navigation of the living archives and ecological considerations: Clumsy-Seeming Mountains, the installation (Tokamachi Snow Center), I think you are likely introducing a new installation, yet you simply state “Our installation was housed…” I think you need to signal to readers that you are dealing with a different installation. Such as: “In the Clumsy-Seeming Mountains installation, …”

**p.7**

 I strongly think that this paragraph is extremely important to your paper and should be included as regular text, not as a footnote. It seems to ground so much of what I keep asking about right here in this paragraph. I would move it up into the paper somewhere. It is a strong paragraph: “we theorize an other-than-human choreography that relativizes the non-human’s non-danceable movement and more-than-human’s non-moving movement. based on Western notions. A key point is the cross-cultural approach that avoids a singular Western notion and offers the clumsy-seemingness which deconstructs a central point of view and proposes more symbiotic and altruistic worldviews and approaches.”

 I think you can remove these couple of words from the photo caption Figure 10: “ *Figure 11 has been*” and start with “Reproduced with permission…”

 “ *composed by Catalina” should probably be changed to “composed by Fernandez.”*

 I think readers will not know what you mean by “slightly ‘off’” Can you give a more tangible experiential descriptor or adjective?

**p.8**

 What sort of dance scores are you referring to on page 8?

 What do you mean by archives? You must tell us what that is. It sounds interesting, but you’ve not told us.

 You wrote “ *By placing MaCA’s archives.*” Typically, in English we would say “ *By placing MaCA’s archives _____ (next to, near, beside, above, juxtaposed with, etc), we aimed to…” *I think you need to qualify what you are doing by adding the right word there.

 I noticed you switch from present to past to present tense in this paragraph. Maybe stay with present tense and speak about it as if it is happening. It will allow readers to experience it as if it is happening for them. I edited these words in your paragraph below: hanging, guide, are, so, can, meanwhile.

 Even though rice fields have been heavily domesticated, they continue to exhibit behaviors that are not solely driven by human desires; rather, they also demonstrate their own performances. Indeed, visitors are aware of the performance of rice fields and watch three huge mountain objects, each about three to five meters in diameter, made by collecting and weaving local kudzu, **hanging** in the space. Mountains made of kudzu in the building and the real mountains outside **guide** the visitors to in-between perspectives. Mountains **are** vessels of organisms and living archives **so** that visitors **can** walk around inside/outside of mountains, **meanwhile **staying close to Earth beings. Thus, the choreography of the clumsy-seeming mountains reopens the discussion of ecological considerations and brings a sense of kinship.

 I think the insertion of the paragraph about the search for second-hand materials and plastic seems oddly placed or even unnecessary. Was this factor a part of your mission or something you simply experienced? If you don’t mention it earlier as part of the project, it comes up as odd on page 8. We are already into your philosophy and mission, and this seems to appear out of nowhere. Do you need it? Maybe earlier you could briefly mention in a method-section about how you managed to use 90% found natural objects in your performance and only the sound and lighting equipment introduced manufactured plastic goods.

 Footnote 14: Does Japan have the “best functioning e-waste management

 System” in the world? I think you left out a modifier.

 Where you wrote “ *he describes*” I think you need to say who said that. There are too many names in the paragraph. I think it’s Yoshida, but it’s hard to follow and I had to check the footnote to be sure.

**p.9**

 Be sure to call Maša only by the surname Buh. First names are never used when speaking respectfully in academic writing.

**Conclusion:**

 You could say, in this case study, we… I thought it was a case study, but now you call it an essay. This is confusing. Isn’t it more of a report about a case study?

 I think your paragraph would be stronger if you dropped out the word “ *effectively”*

*Why qualify your experience when it is more about understanding the process and the relationship?*

**p.11**

 Possibly reconsider changing one of the awareness words here: “awareness of environmental awareness…” Maybe instead state the consciousness of environmental awareness. Or maybe you wish to repeat the word for meaning. In that case keep it.

 Maybe state which decade you are referring to in “over a decade”?

 Before this statement, “At the time of writing,” maybe you could put a heading such as

**Suggestions for Future Research.**

 Ethics and consent:

 You wrote “Ethical approval and consent were not required.” Was this made clear to you through an IRB application. If so, you could say, this study was approved and categorized as exempt upon IRB review.

 I think you have an extra word “that” in here:

*The authors confirm that consent to publish the name of Luz Marina Giraldo.*

*Data Availability:*

*“No data are associated with this article”. It should be “data is”*

**References:**

 For some reason this item is capitalized differently than your other references. Your other references are lowercase.

 Frischkorn M: More-Than-Human Choreography: Handling Things Between

 Logistics and Entanglement. Bielefeld

 Note: I’m not sure it’s needed, but you could put the dates when each of the photographs were taken, if they were taken in a particular year or season of a year.

Is the case presented with sufficient detail to be useful for teaching or other practitioners?

Partly

Is the work clearly and accurately presented and does it cite the current literature?

Partly

If applicable, is the statistical analysis and its interpretation appropriate?

Not applicable

Are all the source data underlying the results available to ensure full access and reuse by other researchers?

Partly

Are the conclusions drawn adequately supported by the data and analysis?

Yes

Is the background of the case’s history and progression described in sufficient detail?

Partly

Reviewer Expertise:

Ph.D. in Dance in Higher Education and Administration Extensive experience publishing qualitative and statistical research about the arts Editor and creator of an online academic, peer-reviewed journal about dance Author of a peer-reviewed book and multiple articles on dance topics Educator of site-specific processes in the arts Experience with dance in South East Asia and Japan.

I confirm that I have read this submission and believe that I have an appropriate level of expertise to confirm that it is of an acceptable scientific standard, however I have significant reservations, as outlined above.
